# Different roles for the acyl chain and the amine leaving group in the substrate selectivity of *N*-Acylethanolamine acid amidase

**DOI:** 10.1080/14756366.2021.1912035

**Published:** 2021-07-13

**Authors:** Andrea Ghidini, Laura Scalvini, Francesca Palese, Alessio Lodola, Marco Mor, Daniele Piomelli

**Affiliations:** a Dipartimento di Scienze degli Alimenti e del Farmaco, Università degli Studi di Parma, Parma, Italy; b Department of Anatomy and Neurobiology, University of California, Irvine, CA, USA; c Department of Pharmaceutical Sciences, University of California, Irvine, CA, USA; d Department of Biological Chemistry and Molecular Biology, University of California, Irvine, CA, USA

**Keywords:** N-acylethanolamine acid amidase, palmitoylethanolamide, substrate selectivity, enzyme kinetic, molecular dynamics

## Abstract

*N*-acylethanolamine acid amidase (NAAA) is an *N*-terminal nucleophile (Ntn) hydrolase that catalyses the intracellular deactivation of the endogenous analgesic and anti-inflammatory agent palmitoylethanolamide (PEA). NAAA inhibitors counteract this process and exert marked therapeutic effects in animal models of pain, inflammation and neurodegeneration. While it is known that NAAA preferentially hydrolyses saturated fatty acid ethanolamides (FAEs), a detailed profile of the relationship between catalytic efficiency and fatty acid-chain length is still lacking. In this report, we combined enzymatic and molecular modelling approaches to determine the effects of acyl chain and polar head modifications on substrate recognition and hydrolysis by NAAA. The results show that, in both saturated and monounsaturated FAEs, the catalytic efficiency is strictly dependent upon fatty acyl chain length, whereas there is a wider tolerance for modifications of the polar heads. This relationship reflects the relative stability of enzyme-substrate complexes in molecular dynamics simulations.

## Introduction

*N*-acylethanolamine acid amidase (NAAA) is a cysteine hydrolase that catalyses the intracellular degradation of the lipid-derived messenger, palmitoylethanolamide (PEA), to palmitic acid and ethanolamine^1^^,^^2^. Biochemical studies have shown that NAAA is primarily expressed in macrophages and B lymphocytes as well as in lungs, spleen and brain^3^^,^^4^. Moreover, immunocytochemical experiments suggest that NAAA is localised to lysosomes^5^. Molecular cloning and sequencing studies have shown that NAAA belongs to the *N*-terminal nucleophile (Ntn) superfamily of enzymes^6^. Like other Ntn proteins, NAAA is translated as a proenzyme and is activated by self-catalysed cleavage which, consistent with the postulated lysosomal localisation of the enzyme, occurs only in acidic conditions^7^. This reaction releases the catalytic nucleophile – Cys126 in humans, Cys131 in mice and rats – and generates two subunits, conventionally termed α and β, which remain physically connected and together shape the enzyme’s substrate-binding site and membrane association loop. The acidic conditions that favour the proteolytic activation of NAAA are also required for the catalytic activity of the enzyme, which shows a maximum at pH 4.5^8^. The X-ray structure of the rabbit enzyme in complex with a molecule of myristic acid strongly suggests that PEA places its 16-carbon saturated chain in the narrow hydrophobic channel delimited by the α and β subunits of NAAA^9^. In this structure, the polar head of PEA accommodates within a solvent exposed cleft comprising the catalytic residue, with the amide group making polar interactions with the oxyanion hole residues (Asn292 and Glu195 in hNAAA) and with the backbone of Asp145. Following this hypothesis, the mechanism of PEA hydrolysis catalysed by NAAA has been recently investigated through QM/MM simulations^10^. According to the proposed mechanism, during the acylation the catalytic cysteine, stabilised by Asp145, acts both as an acid and as a nucleophile, by protonating the amide nitrogen of PEA and favouring the expulsion of the ethanolamine fragment as leaving group upon the formation of a thioester bond. The deacylation would thus occur, following the activation of the deacylating water by the *N*-terminal amino group of the catalytic cysteine, with the carbonyl oxygen stabilised by the interaction with the oxyanion hole residues.

NAAA’s best known substrate, PEA^4^, is a member of the fatty acid ethanolamide (FAE) family of lipid messengers, which also includes anandamide (arachidonoylethanolamide) and oleoylethanolamide (OEA). Anandamide is an endogenous agonist for cannabinoid receptors, the target of Δ^9^-tetrahydrocannabinol in cannabis^11^, while PEA and OEA activate the ligand-operated transcription factor, peroxisome proliferator activated receptor-α (PPAR-α), to regulate energy balance^12–14^, pain^15^, and inflammation^16^^,^^17^. NAAA plays a key role in the control of the cellular levels of PEA. In healthy tissues, this lipid mediator is generated by the action of a structurally unique phospholipase D (*N*-acylphosphatidylethanolamine phospholipase D, NAPE-PLD) that cleaves the glycerophospholipid precursor, *N*-palmitoylphosphatidylethanolamine, to produce FAE and phosphatidic acid^18–20^. PEA formation contributes to tissue homeostasis by recruiting PPAR-α-dependent transcriptional programs that enhance host defence and curb inflammatory responses^21^^,^^22^. Suprathreshold inflammatory stimuli cause a rapid reduction in tissue PEA content, which may enable in turn the development of inflammation^22^. This decline in tissue PEA levels is due to a two-pronged process that involves the suppression of NAPE-PLD transcription^23^ and the enhancement of NAAA expression and activity^24^.

In addition to NAAA, the hydrolysis of PEA is catalysed by fatty acid amide hydrolase (FAAH)^2^. A member of the amidase-signature family of serine hydrolases, FAAH is a transmembrane homodimer found in the endoplasmic reticulum and the nucleus^25^. Structural studies have shown that the relatively large substrate-binding site of this enzyme can accommodate a variety of structurally diverse molecules, including not only FAEs such as anandamide, PEA and OEA, but also amides of long-chain fatty acids with taurine^26^^,^^27^ and glycine^28^. For this reason, the regulatory functions of FAAH are likely to extend beyond the endocannabinoid and PPAR-α systems, to include other as-yet-unknown signalling events mediated by *N*-acyltaurines^29^ and *N*-acylglycines^30^.

In the present study, we used a combination of enzyme kinetic and computational analyses to conduct a systematic investigation of the substrate selectivity of NAAA. The work of Ueda and collaborators has provided evidence for a marked selectivity of NAAA for PEA compared to other *N*-acylethanolamines. More specifically, the relative reactivities of the enzyme were found to be at 100% for PEA, 48% for *N*-myristoylethanolamine, approximately 20% for *N*-stearoylethanolamine and *N*-oleoylethanolamine, 13% for *N*-linoleoylethanolamine and only 8% for anandamide^3^. Building on this work^1^^,^^3^, we show here that the fatty acyl chain of the substrate, rather than its polar head, controls NAAA-mediated catalysis, such that even single-carbon deviations from the optimal 16-carbon saturated chain of PEA markedly decrease the catalytic efficiency. The results provide novel insight into NAAA’s catalytic mechanism and identify PEA as the primary substrate for this enzyme.

## Experimental section

### Preparation of the homology model

The ocNAAA (*Oryctolagus cuniculus*, rabbit NAAA) in complex with a molecule of myristic acid was selected as template structure for homology modelling of rNAAA (rat NAAA). The X-ray structure of ocNAAA and the amino acid sequence of ocNAAA and rNAAA were retrieved from the Protein Data Bank (PDB 6DY1)^9^ and from the Universal Protein Resource (Uniprot ID G1T7U7 for ocNAAA and Q5KTC7 for rNAAA), respectively. Starting from an initial sequence alignment performed with Blast^31^, the homology model was built using the Protein Structure Prediction tool of Prime 5.2^32^ by applying the energy-based method, which allows to build and optimise the residues that do not match the template based on the Prime energy, which accounts for steric, electrostatic and solvation terms^33^. During the procedure, the molecule of myristic acid, the residue of Triton X-100 and the water molecules co-crystalised with ocNAAA were maintained. Once the homology model was built, it was processed through the Protein Preparation tool available in Maestro 11.6^34^^,^^35^. Consistently with the maximum of activity of NAAA observed at pH 4.5, all the histidine and basic residues were modelled in their protonated form. The protonation state for Cys131 and its surroundings was assigned as described in a recent study for our group, where a specific protonation pattern was found to provide the greatest stability during molecular dynamics simulations of human NAAA^10^. Cys131 was modelled in its neutral amine-anionic thiolate form, while the protonation state of acidic residues within 10 Å of the catalytic cysteine was assigned as follows: Asp150, Asp199, Glu200 and Asp304, conserved in both hNAAA and ocNAAA, were modelled in their neutral form. Glu297, corresponding to a lysine residue in hNAAA and ocNAAA, was modelled in its neutral form. All the other acidic residues were modelled in their negatively charged form. The model was then subjected to minimisation using the OPLS3e force field^36^ restraining the position of the heavy atoms to an RMSD of 0.3 Å.

### Docking studies and molecular dynamics simulations

**Figure 1.**
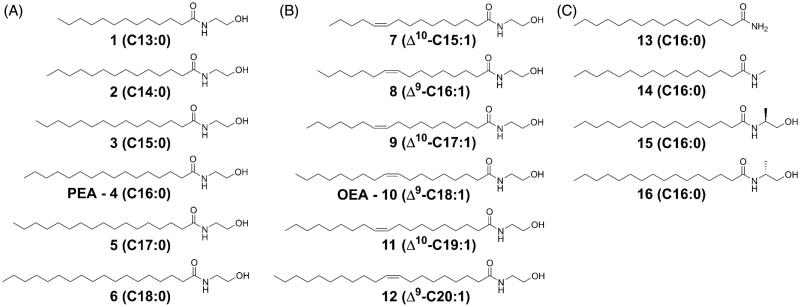
Compounds investigated in the present study: (A) saturated FAEs with C13 to C18 acyl chain (compounds **1**–**6**); (B) monounsaturated FAEs with C15 to C20 acyl chain (compounds **7**–**12**). The Z double bond is in position 9 for even-numbered chains, and in position 10 for odd-numbered chains; C) PEA derivatives carrying modifications on the ethanolamine moiety (compounds **13**–**16**).

Docking studies were performed using Glide 7.9^37^^,^^38^. The docking grid was centred on the centre of mass of residues Cys131, Tyr151, Trp186, Glu200, and Asn292, setting the inner and outer cubic box dimensions to 13 Å and 33 Å, respectively. The substrates were built in Maestro 11.6 and prepared using the Ligprep utility^39^. The van der Waals radii of the protein and of the substrates were reduced by a scaling factor of 0.8 and 0.7, respectively. The standard precision mode was applied for ranking the docking poses. For each substrate, the best docking pose according to the Gscore function was selected, and the corresponding substrate-protein complex was energy-minimised using the OPLS3e force field implemented in Macromodel 12.0^40^ and by applying the Polak-Ribiere conjugate gradient method to a convergence threshold of 0.05 kJ mol^−1 ^Å^−1^. During the minimisation, the atoms of the ligands and of the surrounding residues within 5 Å were free to move, while the backbone of other residues was kept fixed. The resulting complexes were imported in t-leap for the parametrisation with AMBER. For each system, the protein was modelled using the Amberff15ipq force field^41^, while the substrates and the Triton X-100 residue were parametrised using the general amber force field^42^. Each system was solvated using TIP3P water molecules, setting the minimum distance between any atom of the system and the edge of the periodic box to 12 Å. The same procedure was applied to the parametrisation of the ocNAAA-myristic acid model. The pmemd module of the AMBER16 software^43^ was used to run all the molecular dynamics (MD) simulations. Electrostatic and van der Waals interactions were computed within a cut-off of 10 Å, while long-range electrostatic interactions were treated using the particle mesh Ewald (PME) approach^44^. Covalent bonds involving hydrogen atoms were constrained with the SHAKE algorithm and a time-step of 2 fs was applied. All the MD simulations were preceded by an equilibration of 20 ns in NVT conditions and 15 ns in NPT conditions. MD simulations performed starting from the X-ray structure of ocNAAA in complex with myristic acid and from the rNAAA-PEA complex were carried out for 200 ns in NVT conditions at 298 K. This first set of simulations was performed to evaluate the dynamic stability of the homology model compared to the X-ray structure. A second set of simulations comprised MD runs of the complexes of rNAAA with PEA and with the other substrates, performed applying restraints of 0.5 kcal mol^−1 ^Å^−2^ on the Cα atoms of the protein in order to maintain the overall architecture of the protein close to the X-ray structure, and carried out for 30 ns (compounds **1–3**, **5**–**12**, Figure 1) or 200 ns (**4**, **15**, **16**, Figure 1) in NVT conditions at 298 K. Three replicas were performed for each modelled system.

### General information

All chemicals were used as received unless stated otherwise. All reactions were performed under a steady overpressure of nitrogen delivered through a balloon. Thin-layer chromatography (TLC) analysis was conducted on HPTLC aluminium sheets (silica gel 60, F254; Sigma-Aldrich); compounds were visualised by dipping in a solution of *p*-anisaldehyde (2.5% v/v), prepared as follows: 3.7 ml of *p*-anisaldehyde were added to 135 ml of absolute ethanol containing 5 ml of concentrated sulphuric acid and 1.5 ml of glacial acetic acid. The staining solution was stored in a jar covered with aluminium foil at 4 °C. 1H NMR spectra were recorded on a Bruker DRX400 (400 MHz). Chemical shifts (δ scale) are reported in parts per million (ppm), referenced to tetramethylsilane (TMS, 0.00 ppm) or the residual solvent signal (CHCl_3_ in CDCl_3_, 7.26 ppm). 1H NMR spectra are reported in the following order: number of protons, multiplicity and approximate coupling constant (*J* value) in hertz (Hz), and a clear attribution of proton signals is provided. Signal multiplicities were characterised as s (singlet), d (doublet), dd (doublet of doublets), t (triplet), dt (doublet of triplets), q (quartet), quint (quintet) m (multiplet), br (broad signal). 1H NMR spectra are collected in Supporting Information. Mass spectra were recorded on an Agilent 6410 Triple Quad LC/MS system with an ESI interface. The purity of final compounds was assessed by liquid chromatography/tandem mass spectrometry (LC/MS-MS) using an Agilent 6410 Triple Quad system (Agilent Technologies). Prior to analyses, samples were prepared in methanol at a final concentration of 5 µg mL^−1^. Analytes separation was performed on an Agilent Zorbax Eclipse XBD C18 column (2.1 × 50 mm, 1.8 µm particle size) by gradient elution. Flow rate was set at 0.4 ml min^−1^, and the injection volume was 2.0 µL. Solvent A was water and solvent B was methanol, both added of 0.25% v/v acetic acid and 5 mM ammonium acetate. Gradient conditions A: t (0 min): 10% A: 90% B; t (2 min): 5% A: 95% B; t (3 min): 10% A: 90% B. All tested compounds were >95% pure. Specific rotation (${\left[\alpha \right]}_{D}^{20}$) was determined with a Perkin-Elmer 341 polarimeter.

### Synthetic procedures

Compounds **1**–**3** and **5–6** (Figure 1) were synthesised using the following procedure (procedure A; synthetic schemes are collected in the Supporting Information): to an ice-cooled stirring solution of ethanolamine (3.241 mmol) in dichloromethane (6 ml), the fatty acyl chloride (1.621 mmol) was added. A white precipitate appeared immediately, and the reaction was continued with stirring for 60 min. The reaction was quenched with hydrochloric acid (1 M), and the organic phase was washed with a saturated solution of sodium bicarbonate, then water, and brine. The organic layer was treated with magnesium sulphate then filtered and dried under vacuum. The crude product was recrystallised from heptane.

Compounds **4** and **7**–**12** were synthesised using the following procedure (procedure B; synthetic schemes are collected in the Supporting Information): to an ice-cooled stirring solution of the fatty acid (0.725 mmol) and triethylamine (1.813 mmol) in dichloromethane (11 ml), 1-hydroxybenzotriazole (HOBt, 0.798 mmol) and *N,N,N’,N’*-tetramethyl-*O*-(benzotriazol-1-yl)uronium tetrafluoroborate (TBTU, 0.798 mmol) were added and the reaction was stirred for 60 min. Ethanolamine (0.870 mmol) was added. After 2 h the reaction was quenched with hydrochloric acid (1 M), and the organic phase was washed with a saturated solution of sodium bicarbonate, then water, and brine. The organic layer was treated with magnesium sulphate then filtered and dried under vacuum. The crude product was recrystallised from heptane or purified by column chromatography (isocratic elution with a mixture of chloroform:methanol (98:2 v/v)) to give the desired compound.

#### Synthesis of N-(2-hydroxyethyl)tridecanamide (compound 1)

Procedure A, tridecanoyl chloride (0.37 g), ethanolamine (0.19 µL), yield = 405 mg (> 98%) . MS calc.: 257.24, found: 258.3 m/z [M + H]^+^. ^1^H-NMR (400 MHz, Chloroform-*d*): δ 6.17 (br, 1H), 3.71 (t, 2H, *J* = 4.8 Hz), 3.40 (q, 2H, *J* = 5.5 Hz), 3.22 (br, 1H), 2.20 (t, 2H, *J* = 7.6 Hz), 1.63 (pent, 2H, *J* = 7.6 Hz), 1.38–1.14 (m, 18H), 0.88 (t, 3H, *J* = 7.0 Hz).

#### Synthesis of N-(2-hydroxyethyl)tetradecanamide (compound 2)

Procedure A, tetradecanoyl chloride (0.39 g), ethanolamine (0.21 µL), yield = 420 mg (98%) . MS calc.: 271.25, found: 272.3 m/z [M + H]^+^. ^1^H-NMR (400 MHz, Chloroform-d): δ 6.03 (br, 1H), 3.72 (t, 2H, *J* = 5.0 Hz), 3.41 (q, 2H, J = 5.4 Hz), 2.91 (br, 1H), 2.20 (t, 2H, *J* = 7.6 Hz), 1.64 (pent, 2H, *J* = 7.7 Hz), 1.39–1.13 (m, 20H), 0.88 (t, 3H, *J* = 7.1 Hz). Analytical data are consistent with those previously reported in the literature^45^^,^^46^.

#### Synthesis of N-(2-hydroxyethyl)pentadecanamide (compound 3)

Procedure A, pentadecanoyl chloride (0.42 g), ethanolamine (0.20 µL), yield = 437 mg (95%) . MS calc.: 285.27, found: 286.3 m/z [M + H]^+^. ^1^H-NMR (400 MHz, Chloroform-d): δ 6.05 (br, 1H), 3.72 (t, 2H, *J* = 4.9 Hz), 3.42 (q, 2H, *J* = 5.3 Hz), 2.96 (t, 1H, *J* = 5.0 Hz), 2.20 (t, 2H, *J* = 7.4 Hz), 1.63 (pent, 2H, *J* = 7.5 Hz), 1.40–1.13 (m, 22H), 0.88 (t, 3H, *J* = 6.9 Hz). Analytical data are consistent with those previously reported in the literature^45^.

#### Synthesis of N-(2-hydroxyethyl)hexadecanamide (PEA – compound 4)

Procedure B, palmitic acid (0.19 g), TBTU (0.26 g), HOBt (0.11 g), Et3N (250 µL), ethanolamine (53 µL), purified by recrystallisation from heptane, yield = 139 mg 64%. MS calc.: 299.28, found: 300.3 m/z [M + H]^+^. ^1^H-NMR (400 MHz, Chloroform-d): δ 5.97 (br, 1H), 3.72 (t, 2H, *J* = 4.9 Hz), 3.42 (q, 2H, *J* = 5.4 Hz), 2.79 (br, 1H), 2.20 (t, 2H, *J* = 7.5 Hz), 1.63 (pent, 2H, *J* = 7.6 Hz), 1.40–1.15 (m, 24H), 0.88 (t, 3H, *J* = 7.0 Hz). Analytical data are consistent with those previously reported in the literature^45^.

#### Synthesis of N-(2-hydroxyethyl)heptadecanamide (compound 5)

Procedure A, heptadecanoyl chloride (0.48 g), ethanolamine (0.20 µL), yield = 453 mg (87%) . MS calc.: 313.30, found: 314.3 m/z [M + H]^+^. ^1^H-NMR (400 MHz, Chloroform-d): δ 5.96 (br, 1H), 3.72 (q, 2H, J = 4.8 Hz), 3.42 (q, 2H, *J* = 5.4 Hz), 2.75 (t, 1H, *J* = 5.1 Hz), 2.20 (t, 2H, *J* = 7.6 Hz), 1.63 (pent, 2H, *J* = 7.6 Hz), 1.40–1.11 (m, 26H), 0.88 (t, 3H, *J* = 7.0 Hz). Analytical data are consistent with those previously reported in the literature^45^.

#### Synthesis of N-(2-hydroxyethyl)octadecanamide (compound 6)

Procedure A, octadecanoyl chloride (0.49 g), ethanolamine (0.21 µL), yield = 519 mg (98%) . MS calc.: 327.31, found: 328.3 m/z [M + H]^+^. ^1^H-NMR (400 MHz, Chloroform-d): δ 5.89 (br, 1H), 3.80 − 3.65 (m, 2H), 3.43 (q, 2H, *J* = 5.4 Hz), 2.60 (br, 1H), 2.20 (t, 2H, *J* = 7.5 Hz), 1.63 (pent, 2H, *J* = 7.7 Hz), 1.40–1.12 (m, 28H), 0.88 (t, 3H, *J* = 5.9 Hz). Analytical data are consistent with those previously reported in the literature^45^.

#### Synthesis of (Z)-N-(2-hydroxyethyl)pentadec-10-enamide (compound 7)

Procedure B, (*Z*)-pentadec-10-enoic acid (0.17 g), TBTU (0.27 g), HOBt (0.11 g), Et3N (252 µL), ethanolamine (52 µL), purified by column chromatography, yield = 108 mg (53%). MS calc.: 283.25, found: 284.3 m/z [M + H]^+^. ^1^H-NMR (400 MHz, Chloroform-d): δ 5.93 (1H, br), 5.40–5.28 (2H, m), 3.72 (2H, br), 3.42 (2H, q, *J* = 5.4 Hz), 2.67 (1H, br), 2.20 (2H, t, *J* = 7.5 Hz), 2.08–1.88 (4H, m), 1.63 (2H, quint, *J* = 7.5 Hz), 1.40–1.19 (14H, m), 0.89 (3H, t, *J* = 7.0 Hz).

#### (Z)-N-(2-hydroxyethyl)hexadec-9-enamide (compound 8)

Procedure B, (*Z*)-hexadec-9-enoic acid (0.19 g), TBTU (0.27 g), HOBt (0.12 g), Et3N (263 µL), ethanolamine (54 µL), purified by column chromatography, yield = 110 mg (49%). MS calc.: 297.27, found: 298.3 m/z [M + H]^+^. ^1^H-NMR (400 MHz, Chloroform-d): δ 6.01 (1H, br), 5.40–5.28 (2H, m), 3.72 (2H, t, *J* = 4.9 Hz), 3.42 (2H, q, *J* = 5.4 Hz), 2.89 (1H, br), 2.20 (2H, t, *J* = 7.6 Hz), 2.06–1.93 (4H, m), 1.63 (2H, quint, *J* = 7.4 Hz), 1.39–1.17 (16H, m), 0.88 (3H, t, *J* = 7.0 Hz).

#### Synthesis of (Z)-N-(2-hydroxyethyl)heptadec-10-enamide (compound 9)

Procedure B, (*Z*)-heptadec-10-enoic acid (0.18 g), TBTU (0.23 g), HOBt (0.10 g), Et3N (230 µL), ethanolamine (48 µL), purified by column chromatography, yield = 120 mg (58%). MS calc.: 311.28, found: 312.3 m/z [M + H]^+^. ^1^H-NMR (400 MHz, Chloroform-d): δ 6.01(1H, br), 5.40–5.28 (2H, m), 3.72 (2H, t, *J* = 4.9 Hz), 3.41 (2H, q, *J* = 5.4 Hz), 2.89 (1H, br), 2.20 (2H, t, *J* = 7.5 Hz), 2.08–1.91 (4H, m), 1.63 (2H, quint, *J* = 7.5 Hz), 1.40–1.15 (18H, m), 0.88 (3H, t, *J* = 6.7 Hz).

#### Synthesis of (Z)-N-(2-hydroxyethyl)octadec-9-enamide (OEA – compound 10)

Procedure B, (*Z*)-nonadec-10-enoic acid (0.19 g), TBTU (0.23 g), HOBt (0.10 g), Et3N (228 µL), ethanolamine (47 µL), purified by column chromatography, yield = 129 mg (61%). MS calc.: 325.30, found: 326.3 m/z [M + H]^+^. ^1^H-NMR (400 MHz, Chloroform-d): δ 6.03 (1H, br), 5.42–5.26 (2H, m), 3.72 (2H, t, *J* = 4.9 Hz), 3.41 (2H, q, *J* = 5.4 Hz), 2.85 (1H, br), 2.20 (2H, t, *J* = 7.5 Hz), 2.09–1.90 (4H, m), 1.63 (2H, quint, J = 7.5 Hz), 1.48–1.06 ppm (20H, m), 0.88 ppm (3H, t, *J* = 7.0 Hz). Analytical data are consistent with those previously reported in the literature^47^.

#### Synthesis of (Z)-N-(2-hydroxyethyl)nonadec-10-enamide (compound 11)

Procedure B, (*Z*)-nonadec-10-enoic acid (0.21 g), TBTU (0.25 g), HOBt (0.11 g), Et3N (251 µL), ethanolamine (52 µL), purified by column chromatography, yield = 80 mg (33%). MS calc.: 339.31, found: 340.3 m/z [M + H]^+^. ^1^H-NMR (400 MHz, Chloroform-d): δ 5.89 (1H, br), 5.40–5.28 (2H, m), 3.72 ppm (2H, t, *J* = 4.7 Hz), 3.42 ppm (2H, q, *J* = 5.5 Hz), 2.60 (1H, t, *J* = 4.9), 2.20 ppm (2H, t, *J* = 7.5 Hz), 2.08–1.91 (4H, m), 1.63 ppm (2H, quint, *J* = 7.5 Hz), 1.40–1.20 ppm (22H, m), 0.88 ppm (3H, t, *J* = 7.0 Hz).

#### Synthesis of (Z)-N-(2-hydroxyethyl)eicos-9-enamide (compound 12)

Procedure B, (*Z*)-eicos-9-enoic acid (0.23 g), TBTU (0.26 g), HOBt (0.11 g), Et3N (260 µL), ethanolamine (54 µL), purified by column chromatography, yield = 107 mg (41%). MS calc.: 353.33, found: 354.3 m/z [M + H]^+^. ^1^H-NMR (400 MHz, Chloroform-d): δ 5.98 ppm (1H, br), 5.39–5.24 (2H, m), 3.72 ppm (2H, t, *J* = 4.8 Hz), 3.42 ppm (2H, q, J = 5.4 Hz), 2.81 (1H, br), 2.20 ppm (2H, t, *J* = 7.7 Hz), 2.06–1.91 (4H, m), 1.63 ppm (2H, quint, *J* = 7.6 Hz), 1.38–1.18 ppm (24H, m), 0.88 ppm (3H, t, *J* = 7.1 Hz).

#### Synthesis of palmitamide (compound 13)

To a solution of palmitic acid (198 mg, 0.781 mmol) in ice-cold dichloromethane (12 ml), triethylamine (237 mg, 2.344 mmol), HOBt (132 mg, 0.859 mmol) and TBTU (276 mg, 0.859 mmol) were added sequentially, and the mixture was stirred for 30 min. Then ammonium chloride (125 mg, 2.344 mmol) was added to the reaction, that was stirred overnight (synthetic schemes are collected in the Supporting Information). The mixture was then diluted with dichloromethane and washed with HCl 1 M, then saturated NaHCO_3_, and brined. The organic phase was dried over MgSO_4_, and the crude product was recrystallised from heptane to get 133 mg of target product (yield = 67%). MS calc.: 255.26, found: 256.3 m/z [M + H]^+^. ^1^H-NMR (400 MHz, Chloroform-d): δ 5.35 ppm (2H, br), 2.22 ppm (2H, t, *J* = 7.5 Hz), 1.64 ppm (2H, quint, *J* = 7.5 Hz), 1.40–1.14 ppm (24H, m), 0.88 ppm (3H, t, *J* = 7.1 Hz). Analytical data are consistent with those previously reported in the literature^48^.

#### Synthesis of N-methylpalmitamide (compound 14)

To a solution of palmitic acid (210 mg, 0.819 mmol) in ice-cold dichloromethane (12.5 ml), triethylamine (249 mg, 2.457 mmol), HOBt (138 mg, 0.901 mmol) and TBTU (289 mg, 0.901 mmol) were added sequentially, and the mixture was stirred for 30 min. Then methylamine hydrochloride (166 mg, 2.457 mmol) was added to the reaction, that was stirred overnight (synthetic schemes are collected in the Supporting Information). The mixture was then diluted with dichloromethane and washed with HCl 1 M, then saturated NaHCO_3_, and brined. The organic phase was dried over MgSO_4_, and the crude product was recrystallised from heptane to get 139 mg of target product (yield = 63%). MS calc.: 269.27, found: 270.3 m/z [M + H]^+^. ^1^H-NMR (400 MHz, Chloroform-d): δ 5.39 ppm (1H, br), 2.38 (3H, d, *J* = 4.8 Hz), 2.16 ppm (2H, t, *J* = 7.5 Hz), 1.62 ppm (2H, quint, *J* = 7.5 Hz), 1.38–1.15 ppm (24H, m), 0.88 ppm (3H, t, *J* = 7.0 Hz). Analytical data are consistent with those previously reported in the literature^49^.

#### Synthesis of (S)-N-(1-hydroxypropan-2-yl)palmitamide (compound 15)

To a solution of palmitic acid (170 mg, 0.663 mmol) in ice-cold dichloromethane (10 ml), triethylamine (201 mg, 1.989 mmol), HOBt (112 mg, 0.729 mmol) and TBTU (234 mg, 0.729 mmol) were added sequentially, and the mixture was stirred for 30 min. Then (*S*)-2-amino-1-propanol (75 mg, 0.994 mmol) was added to the reaction, that was stirred for 2 more hours (synthetic schemes are collected in the Supporting Information). The mixture was then diluted with dichloromethane and washed with HCl 1 M, then saturated NaHCO_3_, and brined. The organic phase was dried over MgSO_4_, and the crude product was recrystallised from heptane to get 100 mg of target product (yield = 48%). MS calc.: 313.30, found: 314.3 m/z [M + H]^+^. ${\left[\alpha \right]}_{D}^{20}$= −12.3

^1^H-NMR (400 MHz, Chloroform-d): δ 5.53 ppm (1H, br), 4.07 (1H, qd, *J* = 6.7, 3.5 Hz), 3.67 ppm (1H, ddd, *J* = 11.0, 6.2, 3.5 Hz), 3.54 ppm (1H, ddd, *J* = 11.0, 6.2, 4.7 Hz), 2.74 (1H, dd, *J* = 6.2, 4.8 Hz), 2.18 ppm (2H, t, *J* = 7.6 Hz), 1.63 ppm (2H, quint, *J* = 7.4 Hz), 1.38–1.20 ppm (24H, m), 1.17 (3H, d, *J* = 6.9 Hz), 0.88 ppm (3H, t, *J* = 7.0 Hz). Analytical data are consistent with those previously reported in the literature^50^.

#### Synthesis of (R)-N-(1-hydroxypropan-2-yl)palmitamide (compound 16)

To a solution of palmitic acid (153 mg, 0.597 eq) in ice-cold dichloromethane (9 ml), triethylamine (181 mg, 1.790 mmol), HOBt (100 mg, 0.656 mmol) and TBTU (210 mg, 0.656 mmol) were added sequentially, and the mixture was stirred for 30 min. Then (*R*)-2-amino-1-propanol (67 mg, 0.895 mmol) was added to the reaction, that was stirred for 2 more hours (synthetic schemes are collected in the Supporting Information). The mixture was then diluted with dichloromethane and washed with HCl 1 M, then saturated NaHCO_3_, and brined. The organic phase was dried over MgSO_4_, and the crude product was recrystallised from heptane to get 106 mg of target product (yield = 57%). MS calc.: 313.30, found: 314.3 m/z [M + H]^+^. ${\left[\alpha \right]}_{D}^{20}$ = +12.3. ^1^H-NMR (400 MHz, Chloroform-d): δ 5.54 ppm (1H, br), 4.07 (1H, qd, *J* = 6.6, 3.4 Hz), 3.67 ppm (1H, ddd, *J* = 11.0, 6.2, 3.5 Hz), 3.54 ppm (1H, ddd, *J* = 11.0, 6.2, 4.8 Hz), 2.75 (1H, dd, *J* = 6.1, 4.8 Hz), 2.18 ppm (2H, t, *J* = 7.5 Hz), 1.63 ppm (2H, quint, *J* = 7.4 Hz), 1.38–1.20 ppm (24H, m), 1.17 (3H, d, *J* = 6.8 Hz), 0.88 ppm (3H, t, *J* = 7.0 Hz). Analytical data are consistent with those previously reported in the literature^51^.

### Generation of NAAA-overexpressing cells

The encoding sequence of rat NAAA (BC105771) was amplified from the cDNA clone 7930474 (Open Biosystems) using the following primer pair: forward primer 5′- CCCAAGCTTATGGGGACCCCAGCCATCCG (HindIII restriction site is underlined); reverse primer 5′-CGCTCGAGTCA**ATGATGATGATGATGATG**GCTTGGGTTTCTGATC (XhoI restriction site is underlined). A (6x) Histidine tag was introduced in the reverse primer sequence (highlighted in bold). The PCR product was cloned into pCDNA 3.1 vector (Invitrogen) using HindIII and XhoI restriction enzymes (New England Biolabs). HEK-293 cells (American Type Culture Collection) were cultured at 37 °C in an incubator (5% CO_2_) using complete Dulbecco’s modified Eagle’s medium (DMEM) containing 10% foetal bovine serum (FBS), 1% penicillin-streptomycin and 1% L-glutamine, and transfected with the rat NAAA(6xHis)-pCDNA3.1 construct using JetPEI transfection reagent (Polyplus), following manufacturer’s instructions. Stably transfected cells were selected by addition of G418 (1 mg mL^−1^) to the cell culture medium and cell clones were generated by limiting dilution plating. Growing clones were analysed for NAAA expression by western blot (anti-hASAHL, R&D Systems).

### Lysosomal extract preparation

Cell pellets were resuspended in Tris-HCl buffer (50 mM) containing sucrose (0.32 M, pH 7.4). Samples were sonicated with a Branson digital sonifier 102 C, and centrifuged at 800 g for 15 min at 4 °C. The resulting supernatants were centrifuged at 12,000 g for 30 min at 4 °C. The pellets were suspended in phosphate-buffered saline (PBS, pH 7.4) and subjected to two freeze/thaw cycles at −80 °C. The suspensions were centrifuged at 100,000 g for 1 h at 4 °C. Protein concentrations were measured using BCA analysis, and samples were stored at −80 °C until use.

### NAAA activity assay

Substrates were incubated, in a range from 1 to 200 µM, in 100 mM Sodium Phosphate Monobasic, 100 mM Sodium Citrate, 0.1% Triton-X 100, 3 mM DTT, pH 4.5 (NAAA assay buffer) with 4 µg of NAAA at 37 °C for 30 min (triplicate samples). Reactions were terminated by addition of 0.6 ml of a cold mixture of methanol and chloroform (1:1) containing *Z*-10-heptadecenoic acid (for saturated fatty acids, 12.5 ng mL^−1^, Cayman Chemical) or heptadecanoic acid (for monounsaturated fatty acids, 12.5 ng mL^−1^, Nu-Chek Prep) as an internal standard. Samples were centrifuged (1462 g, 15 min, 4 °C), and the organic phases were transferred into new vials, dried and the residues were dissolved in 0.3 ml of methanol:chloroform (9:1, v/v). Fatty acid quantifications were carried out by LC/MS using an Agilent 6410 Triple Quad LC/MS system, and the analytes were eluted from a Zorbax Eclipse XBD-C18 column (2.1 × 50 mm, 1.8 µm pore size, Agilent Technologies) at a flow rate of 0.4 ml min^−1^ for 6 min using a solvent mixture of water (A) and methanol (B), both containing 0.25% acetic acid and 5 mM ammonium acetate (0 min-2 min 90% B, 2 min-3 min 95% B, 3 min-6 min 90% B). Column temperature was set at 40 °C. Electrospray ionisation was in the negative mode and the capillary voltage was 4 kV. N2 was used as drying gas at a flow rate of 13 L min^−1^ and a temperature of 350 °C. The [M−H]− ion was monitored in the selected-ion monitoring (SIM) mode. Calibration curves were generated using commercial fatty acids (Cayman Chemical). NAAA activity was calculated as pmol of fatty acid produced by 1 mg of NAAA in 1 min. Activity data were plotted as function of the substrate concentration, and K_m_ and V_max_ values were calculated using the Michaelis-Menten function analysis on GraphPad Prism version 8.4.2 (GraphPad Software Inc.).

## Results

### Preparation of the NAAA’s substrate library

Figure 1 lists the compounds investigated in the present study as potential substrates for NAAA . These include saturated FAE species with chain length varying from C13 to C18 (Figure 1A), monounsaturated species with chain length varying from C15 to C20 and a double bond in *Z* configuration located at the Δ9 or Δ10 position (Figure 1(B)). PEA derivatives (C16) in which the ethanolamine moiety is variously modified were also evaluated, in order to investigate the effect of the removal of the ethanolamine group or its substitution with bulkier groups (Figure 1(C)). Test compounds were incubated for 30 min in a pH 4.5 buffer (pH optimum for NAAA) containing recombinant rNAAA. The fatty acid products were quantified by liquid chromatography/mass spectrometry (LC/MS) and K_m_ and V_max_ values were determined using standard methods (Table 1)^52^.

**Table 1.** Kinetic parameters for FAE hydrolysis by rat NAAA^a^

Compound	K_m_ (μM)	V_max_ (μM/s)	V_max_/K_m_ (s^-1^)
1	26.28	2.38 × 10^−5^	<1.00 × 10^-6^
2	72.86	1.20 × 10^−3^	1.65 × 10^−5^
3	25.46	8.99 × 10^−4^	3.53 × 10^−5^
4 – PEA	19.81	3.43 × 10^−3^	1.73 × 10^−4^
5	6.54	2.45 × 10^−4^	3.75 × 10^−5^
6	----	----	<1.00 × 10^−6^
7	54.29	6.39 × 10^−4^	1.18 × 10^−5^
8	72.81	9.25 × 10^−4^	1.27 × 10^−5^
9	128.18	7.57 × 10^−3^	5.91 × 10^−5^
10 – OEA	167.28	1.60 × 10^−3^	9.55 × 10^−6^
11	160.95	6.90 × 10^−4^	4.29 × 10^−6^
12	231.70	6.56 × 10^−5^	<1.00 × 10^−6^
13	18.29	5.29 × 10^−3^	2.89 × 10^−4^
14	18.08	8.54 × 10^−3^	4.72 × 10^−4^
15	32.34	1.94 × 10^−2^	5.99 × 10^−4^
16	19.32	4.69 × 10^−3^	2.43 × 10^−4^

^a^ K_m_ and V_max_ were calculated by Michaelis-Menten analysis. In all cases, the standard error of the mean (SEM) was <15% of the mean.

### Acyl chain length role: kinetic studies

**Figure 2.**
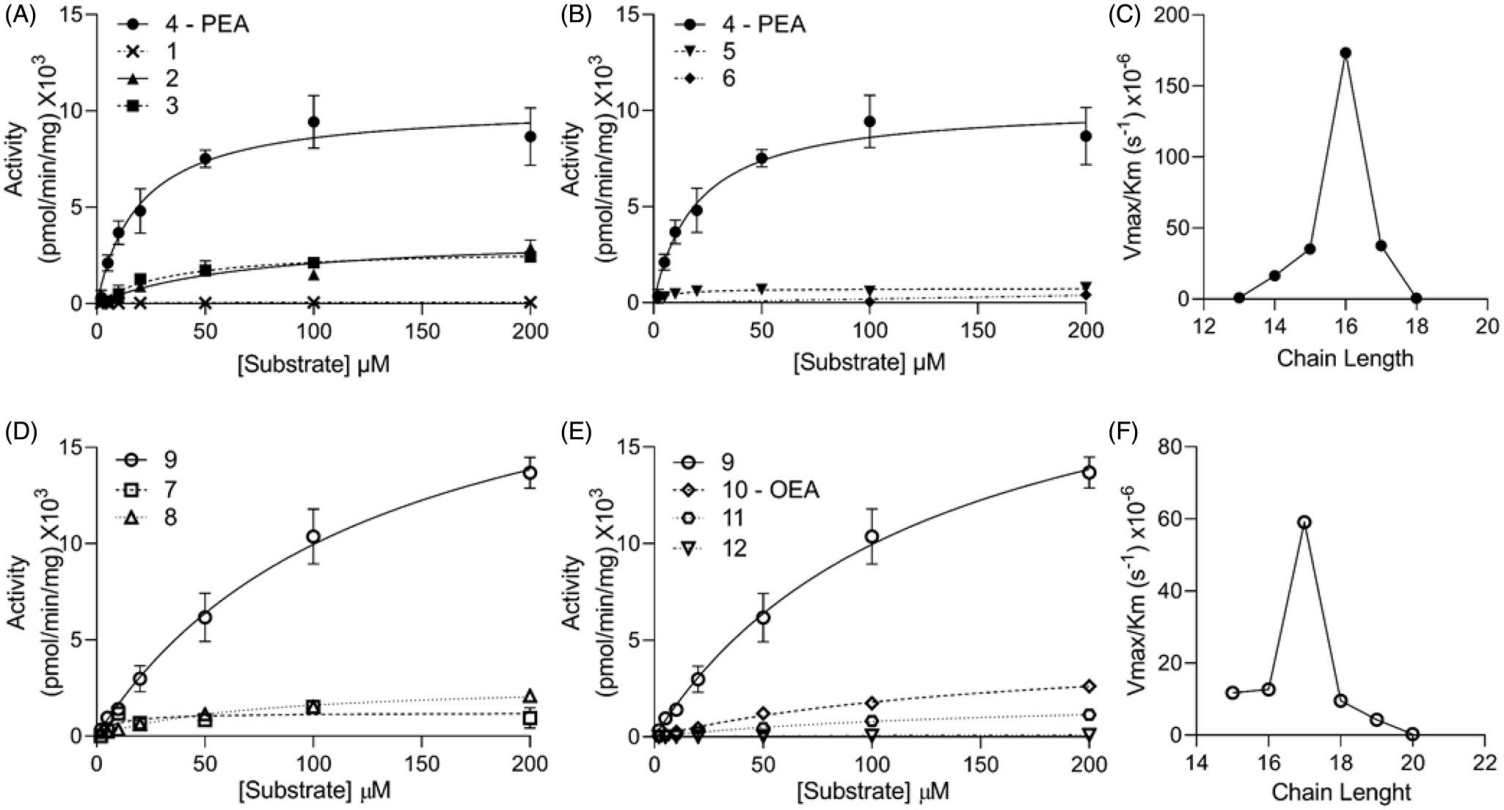
Michaelis-Menten analysis for (A) saturated FAE species with chain lengths shorter than C16 (PEA); (B) saturated FAE species with chain lengths longer than C16; (C) catalytic efficiency (V_max_/K_m_) for saturated FAE species as a function of chain length; (D) monounsaturated substrates with chain lengths shorter than C18; (E) monounsaturated FAE species with chain lengths longer than C18; (F) catalytic efficiency for monosaturated FAE species as a function of chain length. Results are reported as mean ± SEM (each measure was conducted in triplicate).

FAE species with C13, C14 and C15 acyl chains (compounds **1**, **2** and **3**) exhibited lower V_max_ and higher K_m_ values, relative to PEA (C16, compound **4**; Figure 2(A) and Table 1). The V_max_/K_m_ ratio, a measure of the ability of an enzyme to recognise and hydrolyse its substrate, was more than 148-fold higher for PEA than C13 FAE (compound **1**), 10-fold higher than C14 (**2**) and 5-fold higher than C15 (**3**). A similar effect was seen for FAEs with longer acyl chains, *i.e.* C17 (**5**) and C18 (**6**) (Figure 2(B) and Table 1). The hydrolysis product for C18 FAE (**6**), stearic acid, was only detectable at substrate concentrations equal or higher than 100 µM. The results, summarised in Figure 2(C), suggest that NAAA’s catalytic efficiency towards saturated FAE species is maximal for PEA (compound **4**, C16) and sharply declines when the fatty acyl chain is either shortened (**1**–**3**, C13–15) or lengthened (**5**–**6**, C17–18).

We next asked whether the introduction of a *cis* double bond might affect the efficiency of NAAA-mediated FAE hydrolysis. We prepared and tested FAE species with acyl chains C15:1Δ10 (**7**), C16:1Δ9 (**8**), C17:1Δ10 (**9**), C18:1Δ9 (OEA, **10**), C19:1Δ10 (**11**) and C20:1Δ9 (**12**) (Figure 2(D,E)). FAE C17:1Δ10 was the best NAAA substrate in this series (Figure 2(D,E)) and displayed a catalytic efficiency that was superior to both shorter-chain (**7**, **8**) and longer-chain compounds (**10**–**12**) including OEA (Figure 2(F)). Of note, all monounsaturated FAEs exhibited significantly lower V_max_/K_m_ ratios compared to PEA (Table 1).

### Polar head role: kinetic studies

**Figure 3.**
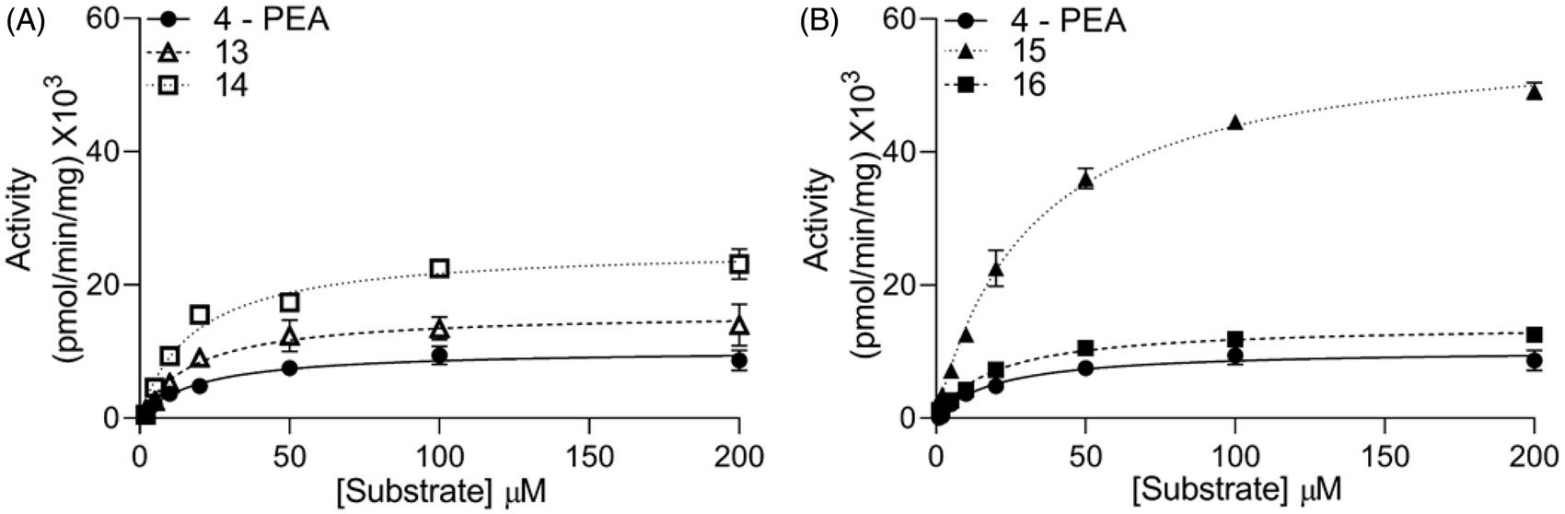
Michaelis-Menten analysis (A) for PEA derivatives lacking the hydroxyl group, and (B) hindered stereoisomers PEA derivatives. Results are shown as mean ± SEM (each measure was conducted in triplicate).

The polar head of PEA occupies a large cavity of the substrate-binding site, which is oriented towards the solvent. To determine whether substitutions in this moiety might affect catalytic efficiency, we prepared four PEA derivatives in which the ethanolamine fragment was replaced with: *i*) a hydrogen atom, yielding the primary amide **13**; *ii*) a methylamine group, yielding the secondary amide **14**; or *iii*) a 2-amino-1-propanol group in the (*S*) or (*R*) configuration (**15** and **16**, respectively). All the substrates were hydrolysed by NAAA, albeit with different V_max_ and K_m_ (Table 1). Of note, NAAA hydrolysed compounds **13** and **14** faster than what observed for PEA and with comparable K_m_ values, suggesting that the FAE terminal hydroxyl group is dispensable for catalysis (Figure 3(A) and Table 1). Moreover, the kinetic parameters for the 2-amino-1-propanol derivative **16** were comparable to those of PEA, whereas the V_max_ of its enantiomer **15** was approximately 6-fold higher (Table 1 and Figure 3(B)).

### NAAA homology model and docking of PEA

**Figure 4.**
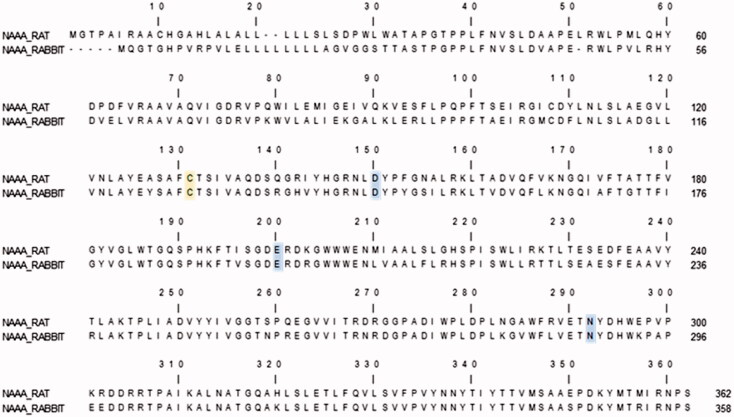
Sequence alignment between the rat and rabbit forms of NAAA. The catalytic cysteine residues (corresponding to Cys131 in rat and Cys127 in rabbit) are highlighted in yellow. Other residues involved in catalysis are highlighted in cyan.

To investigate the molecular basis of the substrate recognition and specificity of rNAAA, we built a homology model of the enzyme starting from the available crystal structure of the rabbit form of the enzyme (*Oryctolagus cuniculus*, ocNAAA) in complex with a molecule of myristic acid (C14:0). The selection of the template structure was driven by the fact that myristic acid is a natural product of NAAA catalysis and is thus unlikely to cause significant perturbations to the co-crystallised lipophilic pocket, compared to the perturbation expected for reversible or irreversible inhibitors such as ARN19702 and ARN726^9^. Rat and rabbit NAAA share 80% sequence identity and, most significantly, the residues that line the hydrophobic channel and the cavity that carries the residues involved in the catalytic mechanism are highly conserved in both the forms of the enzyme (Figure 4)^9^.

**Figure 5.**
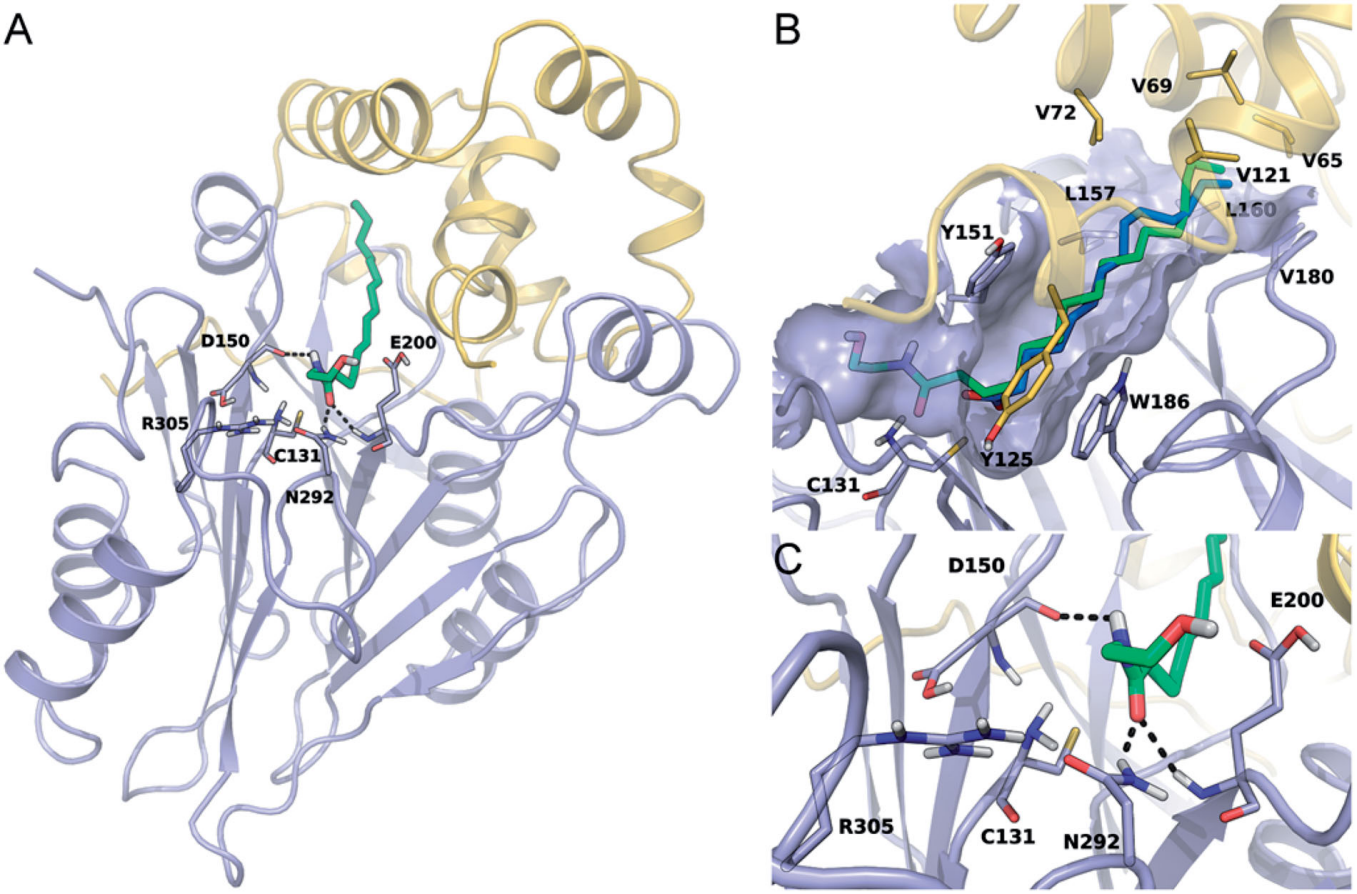
(A) Docking pose of PEA (green carbon atoms) in the homology model of rNAAA developed in the present study. The substrate is housed in a binding site shaped by the enzyme’s α and β subunits (yellow and light blue ribbons, respectively). (B) Side-view of the binding site of NAAA, with detail of its substrate-binding pocket. Hydrophobic residues of the α subunit (Val65, Val69, Val72, Val121 and Tyr125; yellow carbon atoms and ribbons) and the β subunit (Tyr151, Leu157, Leu160, Val180 and Trp186, light blue carbon atoms and ribbons) line a narrow and relatively straight channel occupied, in the crystal structure of ocNAAA, by a myristic acid molecule (blue carbon atoms). (C) Detail of the catalytic site of the enzyme. The catalytic residue Cys131 is placed in a wide cavity, lined by Asp150, Glu200, Asn292 and Arg305. The amide group of PEA makes polar interactions with the CO group of Asp150 backbone, and with the NH group of Glu200 and the side chain of Asn292. The hydroxyethyl moiety is oriented towards the solvent.

We modelled the catalytic cysteine in its neutral amine-anionic thiolate form and the adjacent aspartic acid (Asp150) with its carboxylic group in neutral state and engaged in an H-bond with the neutral form of the enzyme’s *N*-terminal amine. The assignment of the protonation state of ionisable residues was driven by the results of our previous work showing that a supervised proton distribution is fundamental for the dynamic stability of hNAAA^10^. The catalytic site and its surroundings are highly conserved in human, rabbit and rat NAAA, and the protein stability likely relies on a proton distribution within the catalytic residue and the acid residues in proximity of the active site that is similar across different species. PEA was docked within the energy-minimised model of rat NAAA (Figure 5(A)). In this model, the palmitoyl chain of PEA occupies a linear and narrow hydrophobic channel defined by the side chains of Tyr151, Leu157, Leu160, Val180 and Trp186 of the β subunit, and Val65, Val69, Val72, Val121 and Tyr125 of the α subunit. This is the same channel occupied by the fatty acyl tail of myristic acid co-crystallised in rabbit NAAA (Figure 5(B))^9^. The polar head of PEA is located at the entrance of the channel with its amide group placed in proximity of the Cys131 thiolate and pointing towards the backbone carbonyl of Asp150, and its carbonyl moiety within the oxyanion hole formed by the backbone NH of Glu200 and the side chain of Asn292. The hydroxyl group of PEA lies in the solvent-exposed cavity that is lined by the terminal amino group of Cys131, and by the side chains of Glu200, Asn292 and Arg305 (Figure 5(C)).

### Evaluation of the model stability by molecular dynamics simulations

**Figure 6.**
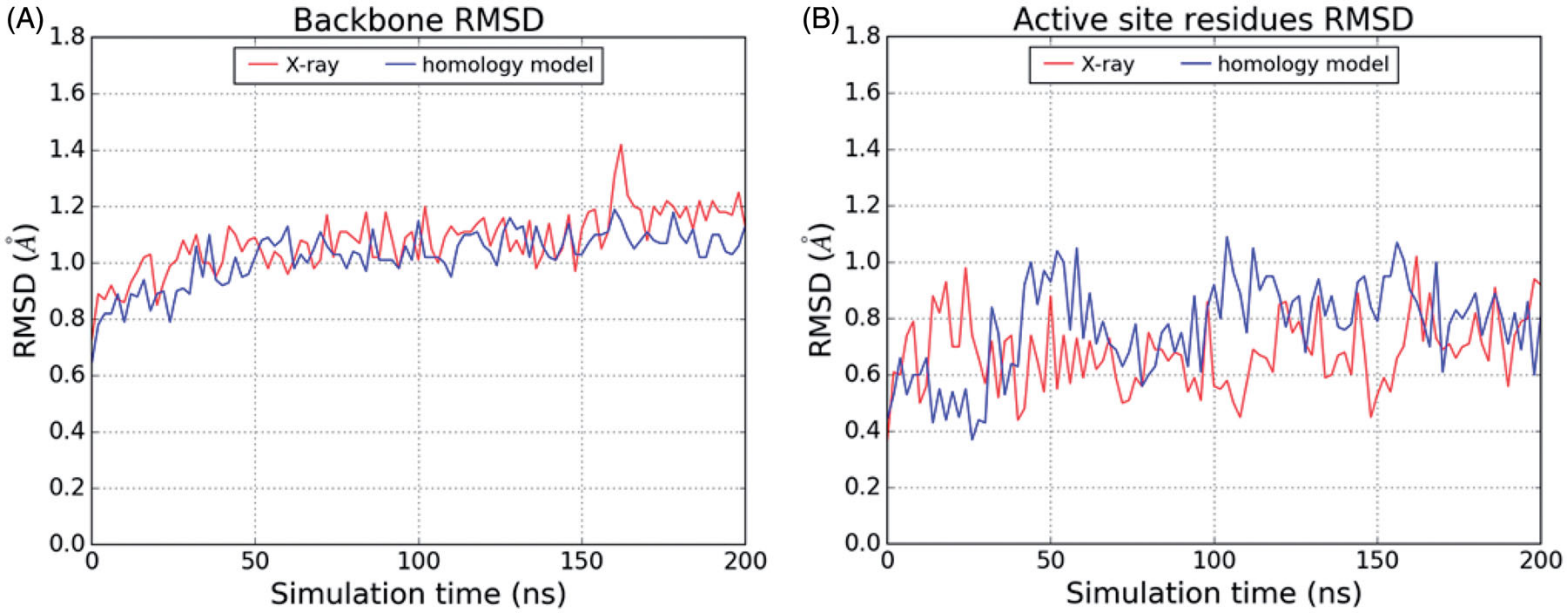
RMSD analysis for the backbone atoms (A) and for the heavy atoms of the residues shaping the active site (comprising Cys131, Asp150, Glu200, Asn292 and Arg305, (B) of the crystallographic structure of ocNAAA in complex with myristic acid (red lines) and of the homology model of rNAAA modelled in complex with PEA (blue lines).

We submitted the rNAAA-PEA complex to a 200 ns molecular dynamics simulation in order to assess its dynamic stability. The analysis of root mean squared deviation (RMSD) of the protein backbone and of the heavy atoms of residues that shape the active site revealed that the overall geometry of the model is conserved, with the RMSD fluctuating around an average value of 1.08 Å. A similar value was recorded in a simulation performed starting from the template structure of the crystallised ocNAAA-myristic acid complex (Figure 6(A)). RMSD analysis for the heavy atoms of Cys131, Asp150, Glu200, Asn292 and Arg305, which shape the catalytic site and the cavity that accommodates the polar head of the substrate, revealed that the active site architecture is also stable (Figure 6(B)), confirming that the model is suitable for molecular modelling studies.

### Acyl chain role: molecular modelling studies

**Figure 7.**
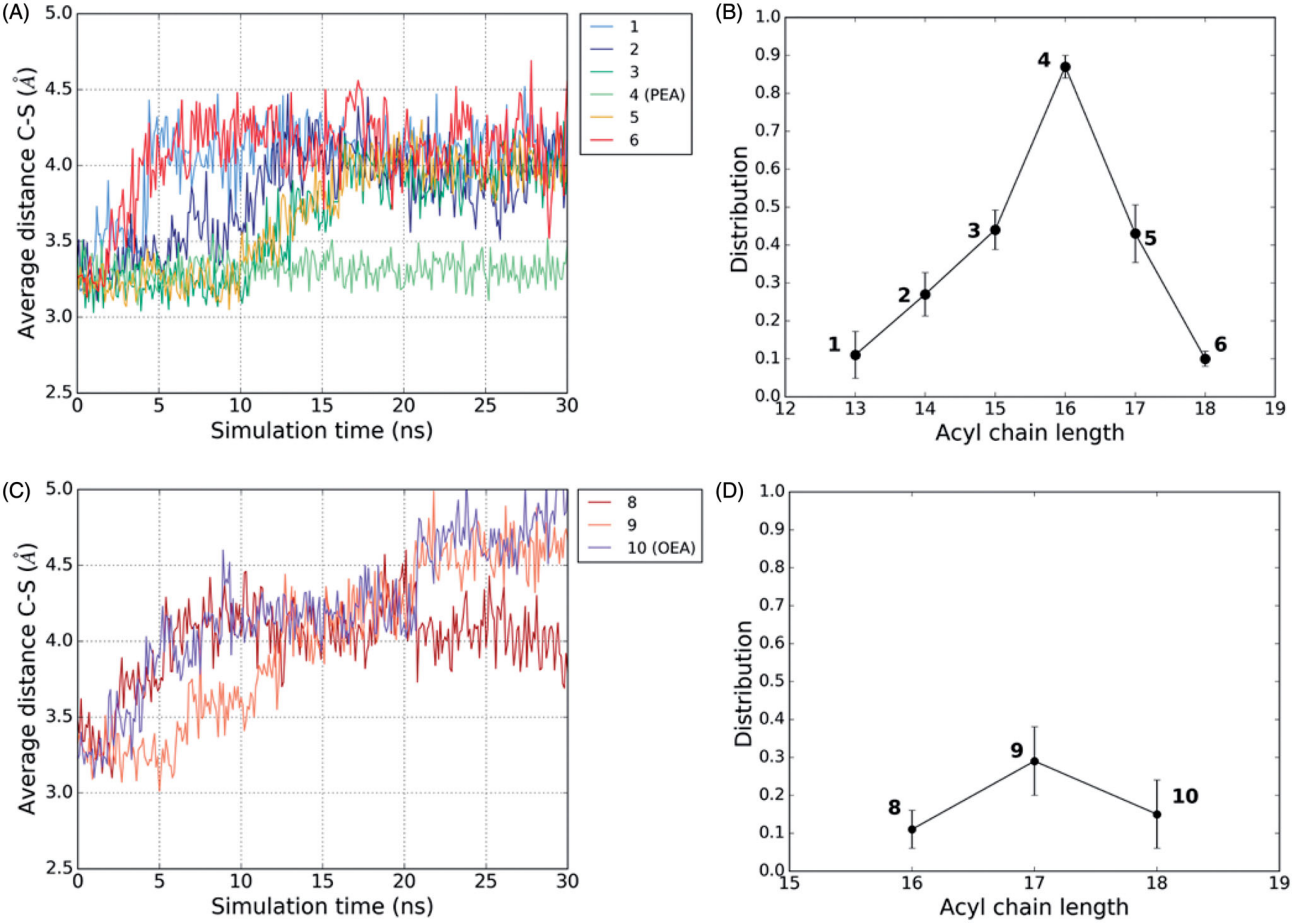
Evolution during MD simulations of the interatomic distance between the sulphur atom of Cys131 and the carbonyl carbon of compounds **1**–**6** and (A). The average value of the C-S distance recorded during MD simulations (three replicas performed) is reported for each compound. Analysis of the distribution of the pro-reactive distance between the carbonyl carbon of the substrates and the sulphur atom of Cys131 reported as the portion of MD simulation frames in which this distance assumes a value lower than 3.5 Å. The average value measured for three 30 ns MD simulations is reported (B). Analyses of the evolution of C-S distance and (C) and of the distribution of the pro-reactive distance (D) are reported also for three replicas of MD simulations of rNAAA complexes with compounds **8**–**10**.

We used the computational model described above to evaluate the effects of acyl-chain substitutions on the dynamic stability of the substrate-NAAA complex. In a series of MD simulations, we recorded the distance between the FAE carbonyl carbon and the sulphur atom of Cys131 as a diagnostic measurement of the complex stability and of the enzyme’s propensity to hydrolyse the substrate^10^. The analyses revealed that the NAAA-PEA complex was characterised by high dynamic stability, with the acyl chain enclosed within the hydrophobic channel of the enzyme and the amide group steadily placed within the active site. By contrast, FAEs with either shorter or longer acyl chains showed a higher degree of mobility. In particular, measuring the distance between the carbonyl carbon of the substrates and the sulphur atom of Cys131 as a function of time, we found that compound **1** formed an unstable complex in which the amide group easily moved away from the active site, losing the possibility to engage the carbonyl carbon and the sulphur atom of Cys131 in interactions that would favour the nucleophilic attack (Figure 7(A)). Similarly, compound **6** could not maintain the initial conformation, with the amide head in the active site, for more than 2.5 ns. On the other hand, compounds **2**, **3** and **5** showed an intermediate behaviour in that they formed complexes that were more stable than **1** or **6** but less stable than PEA (Figure 7(A)).

Our experimental results suggest the existence of a close correlation between the catalytic efficiency of NAAA and the length of its substrate acyl chain (Figure 1). To gain insights into the structural basis for this finding, for each compound we reassessed the distance between the carbonyl carbon of the substrates and the sulphur atom of Cys131 in a set of three 30-ns MD simulations. We considered the average value of the portion of the total amount of MD simulation frames in which the distance between the carbonyl carbon of the substrates and the sulphur atom of Cys131 is shorter than 3.5 Å in the three simulations performed for each substrate. This approach allowed us to correctly rank this set of molecules according to their propensity to be hydrolysed by NAAA (Figure 7(B)). The same procedure was applied to investigate the interaction between NAAA and monounsaturated FAEs. Starting from the results obtained for the saturated substrates, showing a higher instability for compounds bearing even a single-carbon deviation from the ideal 16-carbon chain of PEA, we hypothesised that a similar trend would characterise the behaviour of monounsaturated substrates. We thus focussed on the rationalisation of compound **9**, which shows the highest catalytic efficiency among the unsaturated substrates, and of compounds **8** and **10**. Notably, the presence of an unsaturation in position Δ9 (compounds **8** and **10**) or Δ10 (compound **9**), did not affect the binding mode of these substrates, which similarly to what observed for compounds **1–6** placed the amide group within the active site, taking H-bonds with the oxyanion hole residues and the backbone of Asp150. The position of the *cis* double bond of the three compounds slightly overlapped, fitting the same portion of the hydrophobic channel occupied by the piperazine fragment of ARN19702 in the X-ray structure of hNAAA (PDB: 6DXX.pdb)^9^ and ocNAAA (PDB: 6DXZ.pdb)^9^ in complex with the non-covalent inhibitor. For all such substrates the starting binding mode was generally characterised by high mobility. Compound **9**, that carries the unsaturation at position Δ10, was characterised by a higher stability compared to **8** and **10**. Nonetheless, compound **9** could not maintain the initial conformation, with the amide group inserted within the active site, for more than 10 ns (Figure 7(C)). Similar to what seen with saturated FAEs, the MD simulations were in good agreement with the experimental results, and correctly ranked these derivatives (Figure 7(D)). This approach, however, was not able to capture the improvement of the V_max_/K_m_ ratio from compound **5** to its unsaturated analogue **9**, which showed a lower propensity to maintain a stable complex compared to compound **5**. A similar discrepancy between the MD simulations and the experimental results was also observed for compound **10**, which is characterised by a slightly improved V_max_/K_m_ ratio compared to its saturated analogue **6**, while both **10** and **6** could not maintain the amide group within the active site for more than 5 ns. Taken together, these results suggests that the introduction of a *cis* double bond at the Δ10 position of the 17-carbon chain derivative and, although to a lower extent, a the Δ9 position of the 18-carbon chain derivative, would improve the catalytic efficiency. On the other hand, while this computational protocol based on the MD simulations correctly ranks derivatives within the same class of substrates, it is not efficient in the rationalisation of variations in the catalytic efficiency values across the saturated and unsaturated classes of NAAA substrates.

### Polar head role: modelling studies

**Figure 8.**
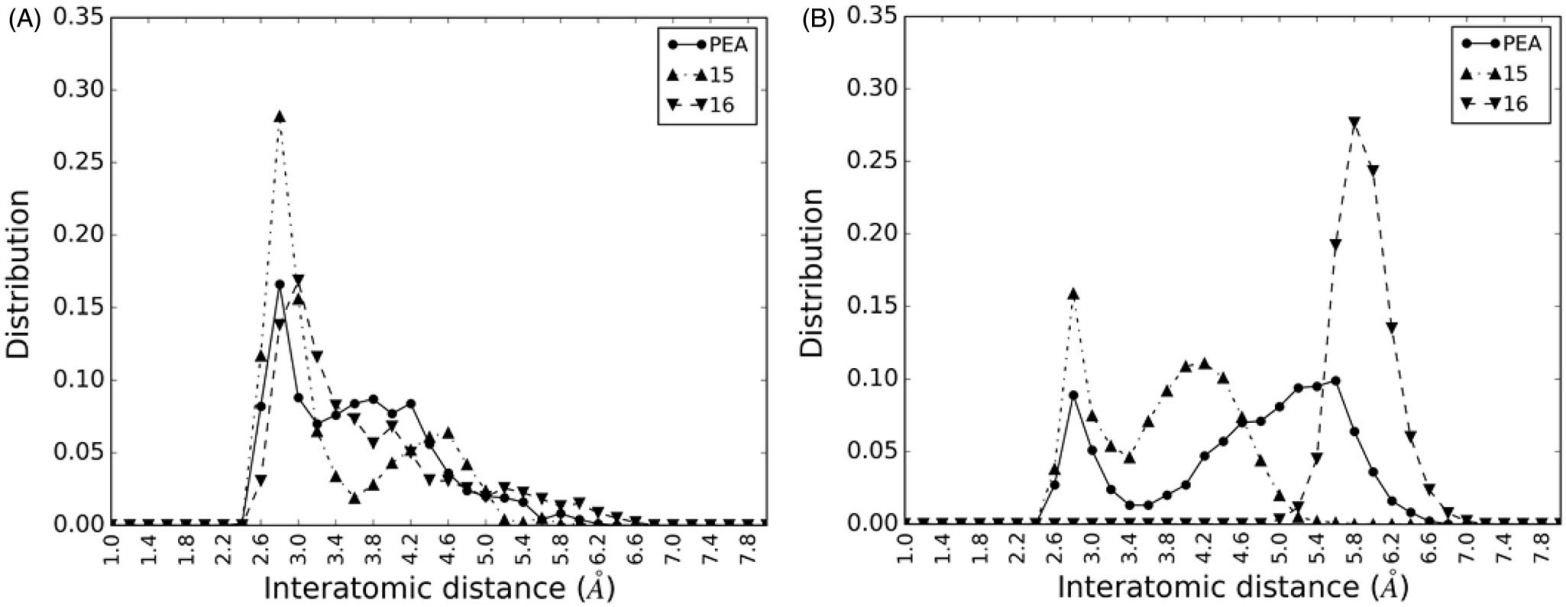
Distribution of the interatomic distances between the hydroxyl group of PEA, **15** and **16**, and the oxygen atom of the Asp150 backbone carbonyl (A) or the amino nitrogen of Cys131 (B). Distances lower than 3 Å reveal strong polar interactions between the two groups.

To investigate the effect of modifications at the ethanolamide portion, and specifically of the replacement of the hydroxyl group with a bulkier fragment, we performed a set of 200 ns MD simulations of the complexes between NAAA and *S*- and *R*-methyl derivatives of PEA (**15** and **16**, respectively), and compared the results with an analogous simulation on the NAAA-PEA complex. The simulations showed that both **15** and **16**, similarly to PEA, are characterised by high stability, with the amide fragment steadily maintained within the active site and the carbonyl carbon at a distance from the sulphur atom of Cys131 oscillating around an average value of ≈3.3 Å, which is consistent with a nucleophilic attack. While the values recorded indicated that all the three substrates maintain a pro-reactive accommodation within the active site, they did not account for the higher propensity of **15** to be hydrolysed by NAAA relative to its *R*-enantiomer and PEA. For this reason, we analysed the MD simulations focussing on the behaviour of the hydroxyl group of the polar head in the three substrates. The plots reported in Figure 8 show the frequency distributions of the polar interactions between the substrate hydroxyl group and the CO moiety of the Asp150 backbone (Figure 8(A)) or the *N*-terminal amino group of Cys131 (Figure 8(B)), measured as the interatomic distances between the heavy atoms recorded throughout the 200-ns MD simulations. The distributions showed that the interatomic distances between the oxygen atoms of the hydroxyl fragment and the CO group of Asp150 were frequently lower than 3 Å. The frequency of low distance-conformations was significantly higher with **15**, compared to PEA and **16**. An additional polar interaction between the hydroxyl group and the terminal amino group of Cys131 was also observed for PEA and **15**, with conformations having distances shorter than 3 Å more populated for the latter.

## Discussion

In this study, we combined biochemical and molecular modelling approaches to delineate a detailed outline of NAAA’s substrate selectivity. This is critical to define the precise contribution of this enzyme to lipid-amide signalling and may be helpful in the design of better inhibitors. Starting from a library of potential substrates, we determined the K_m_ and V_max_ values, and used the V_max_/K_m_ ratio as a parameter to evaluate catalytic efficiency. The results of kinetic experiments were rationalised by evaluating the dynamic stability of the complexes between the substrates and a model of rNAAA.

Our results show the existence of a strict correlation between the catalytic efficiency of the enzyme and the length of its substrate acyl chain, with an optimum for PEA. A steep fall in the V_max_/K_m_ ratio of substrates with slightly different acyl chains suggests that the 16 carbon-atom saturated acyl chain of PEA is a critical feature for the recognition of the substrate and its hydrolysis. This substrate selectivity mirrors the peculiar shape of NAAA’s binding site, characterised by a straight and narrow hydrophobic channel. The correlation between the acyl chain length and the catalytic efficiency was also reflected by the results of molecular modelling studies. MD simulations results suggest that NAAA selectively recognises substrates able to fit within its narrow and straight binding pocket, while substrates with a non-optimal acyl chain form unstable complexes in which the amide group is not allowed to take the interactions that would favour the nucleophilic attack. Lastly, we investigated the role of the ethanolamine group by testing palmitamide, *N*-methylpalmitamide, and *R-* and *S-*methyl PEA. Kinetic experiments show that derivatives lacking the ethanolamine portion are rapidly hydrolysed by NAAA, with a K_m_ comparable to that measured for PEA. Consistently with these data, we recently reported the results of theoretical studies on the mechanism of NAAA showing that the acylation step modelled for PEA and *N*-methylpalmitamide is characterised by a similar free-energy profile^10^. These results thus further support the hypothesis that the ethanolamine head has little or no role in the hydrolytic process catalysed by NAAA. The experimental results obtained for *R-* and *S-*methyl PEA, which showed K_m_ values comparable to PEA, also pointed out that substrates with relatively bulky polar heads can be accommodated within the active site of the enzyme. MD simulations suggest that substitution in the α position of the ethanolamine group promotes a conformational selection in the substrates, promoting an enrichment in conformations that can make polar contacts within the active site of the enzyme for the *S* enantiomer. Collectively, the results of our biochemical and molecular modelling studies outline the fundamental role of the hydrophobic tail of substrates in the recognition process by NAAA, suggesting that the optimisation of a hydrophobic portion able to mimic the conformation of the 16-carbon saturated tail of PEA would be a critical step in the design of novel site-active NAAA inhibitors.

## Supplementary Material

Supplemental Material
