# Supplementary material for: Different roles for the acyl chain and the amine leaving group in the substrate selectivity of N-Acylethanolamine acid amidase
Source: J Enzyme Inhib Med Chem. 2021 Jul 13;36(1):1411–23. doi: 10.1080/14756366.2021.1912035 (PMC8279155; doi:10.1080/14756366.2021.1912035)

Supporting information for:

Different roles for the acyl chain and the amine leaving group in the substrate selectivity of *N*-Acylethanolamine Acid Amidase.

Andrea Ghidini,[a#] Laura Scalvini,[a#] Francesca Palese,[b] Alessio Lodola,[a]

Marco Mor,[a] and Daniele Piomelli [*b,c,d]

[a] Dipartimento di Scienze degli Alimenti e del Farmaco, Università degli Studi di Parma, Parco Area delle scienze 27/A, I- 43124 Parma, Italy

[b] Department of Anatomy and Neurobiology, University of California, Irvine, California 92697-4625

[c] Department of Pharmaceutical Sciences, University of California, Irvine, California 92697-4625

[d] Department of Biological Chemistry and Molecular Biology, University of California, Irvine, California 92697-4625

NMR spectra

N-(2-hydroxyethyl)tridecanamide (1)

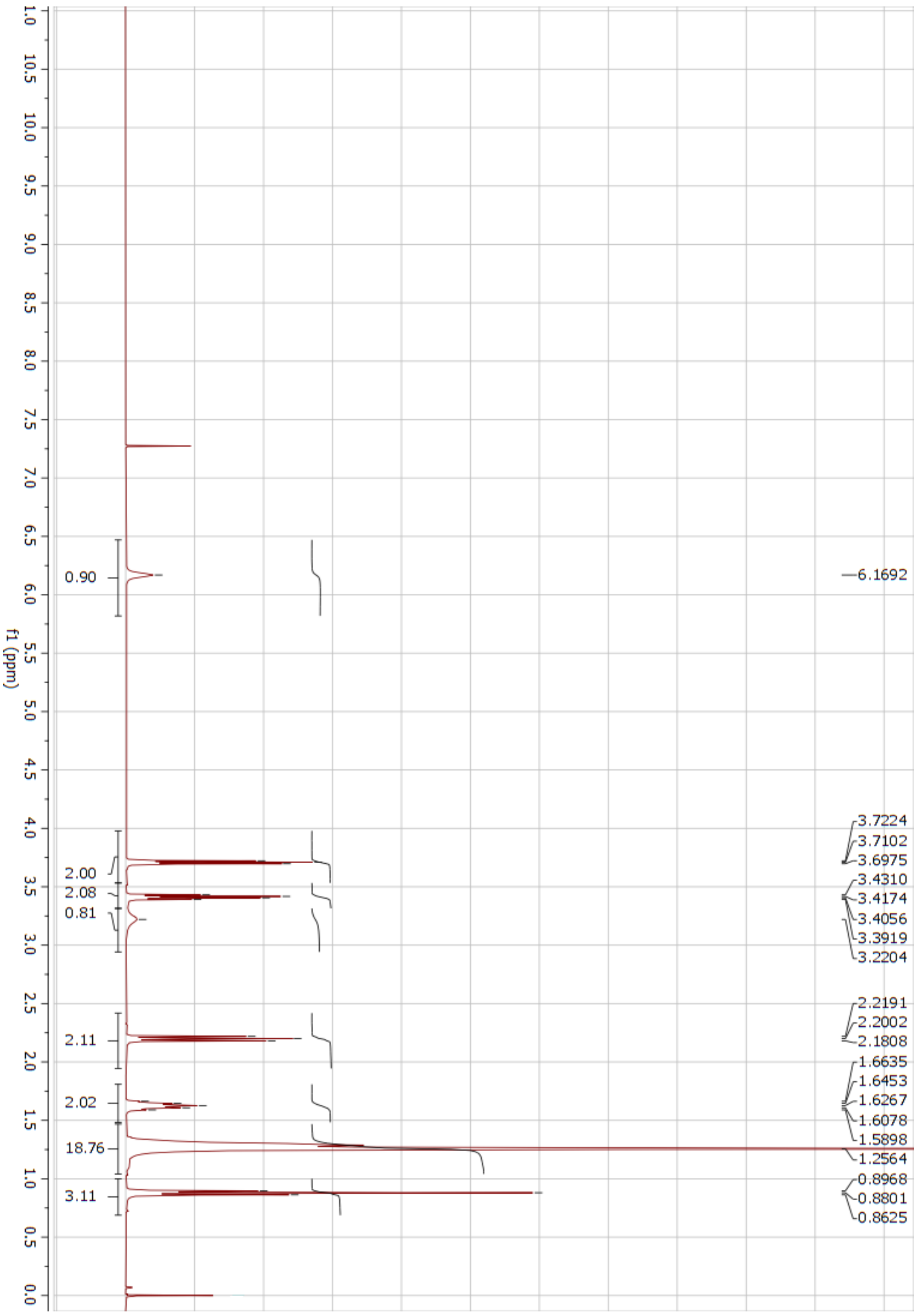

***N*-(2-hydroxyethyl)tetradecanamide (2)**

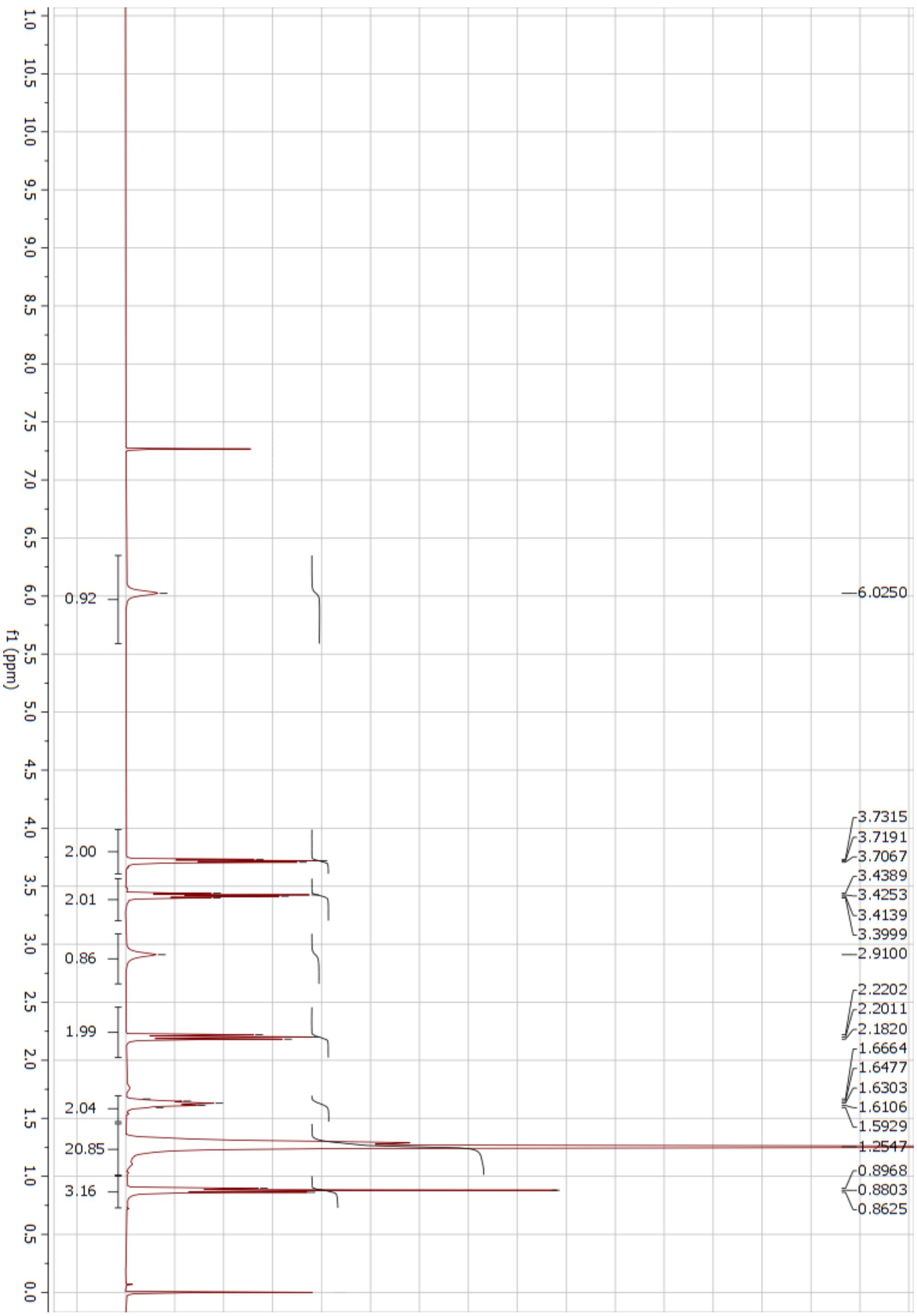

***N*-(2-hydroxyethyl)pentadecanamide (3)**

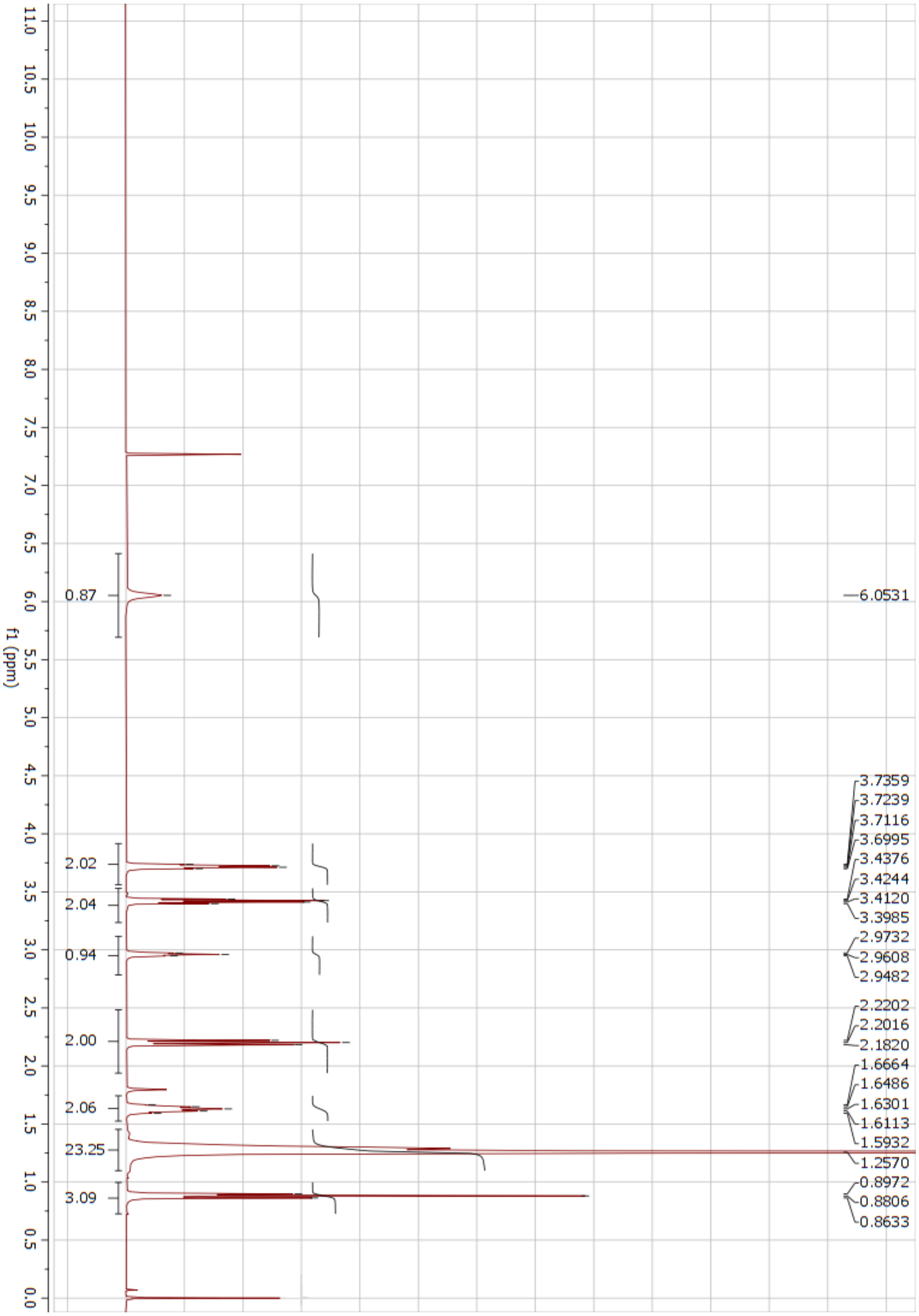

***N*-(2-hydroxyethyl)hexadecanamide (PEA - 4)**

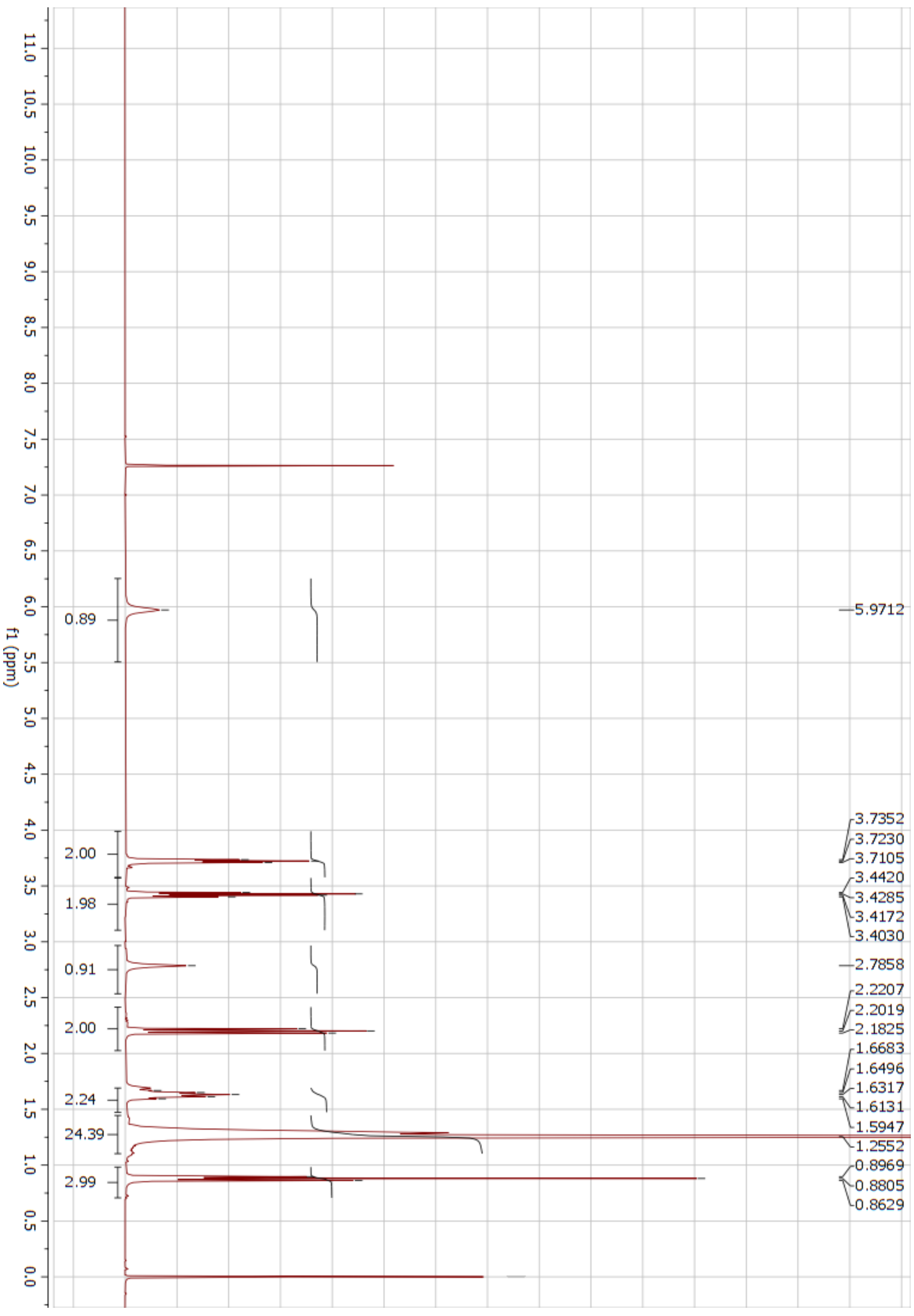

N-(2-hydroxyethyl)heptadecanamide (5)

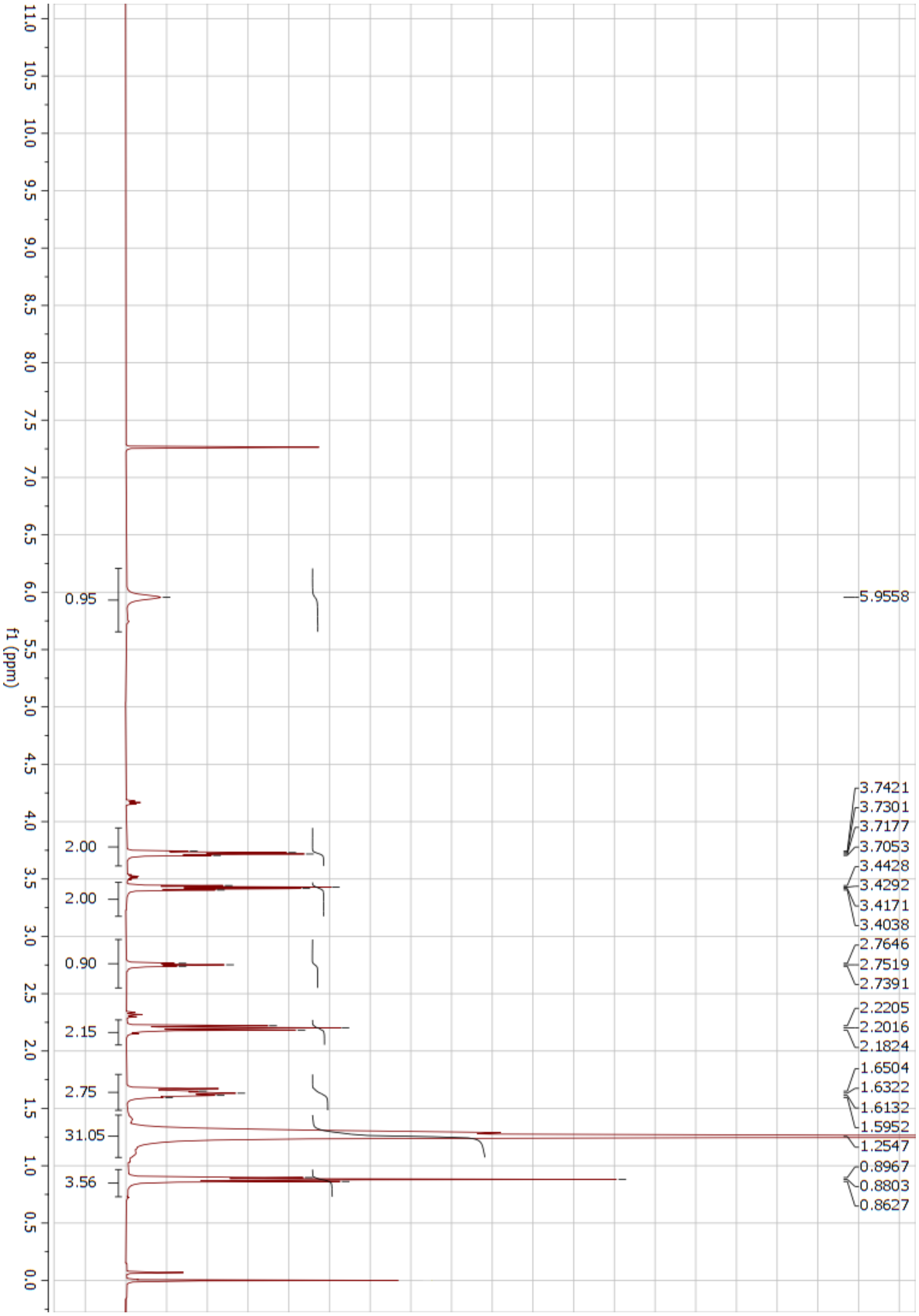

***N*-(2-hydroxyethyl)octadecanamide (6)**

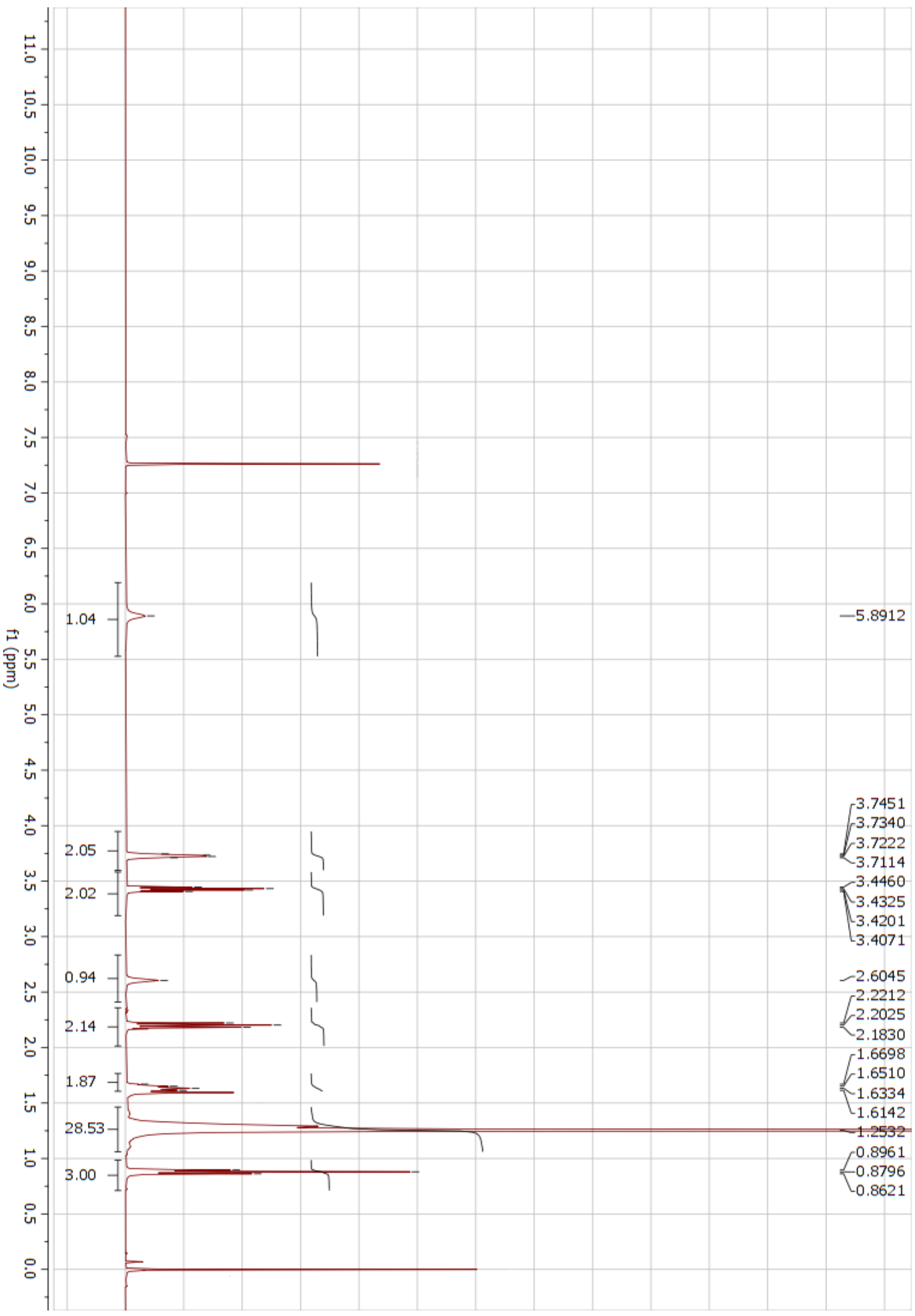

(Z)-*N*-(2-hydroxyethyl)pentadec-10-enamide (7)

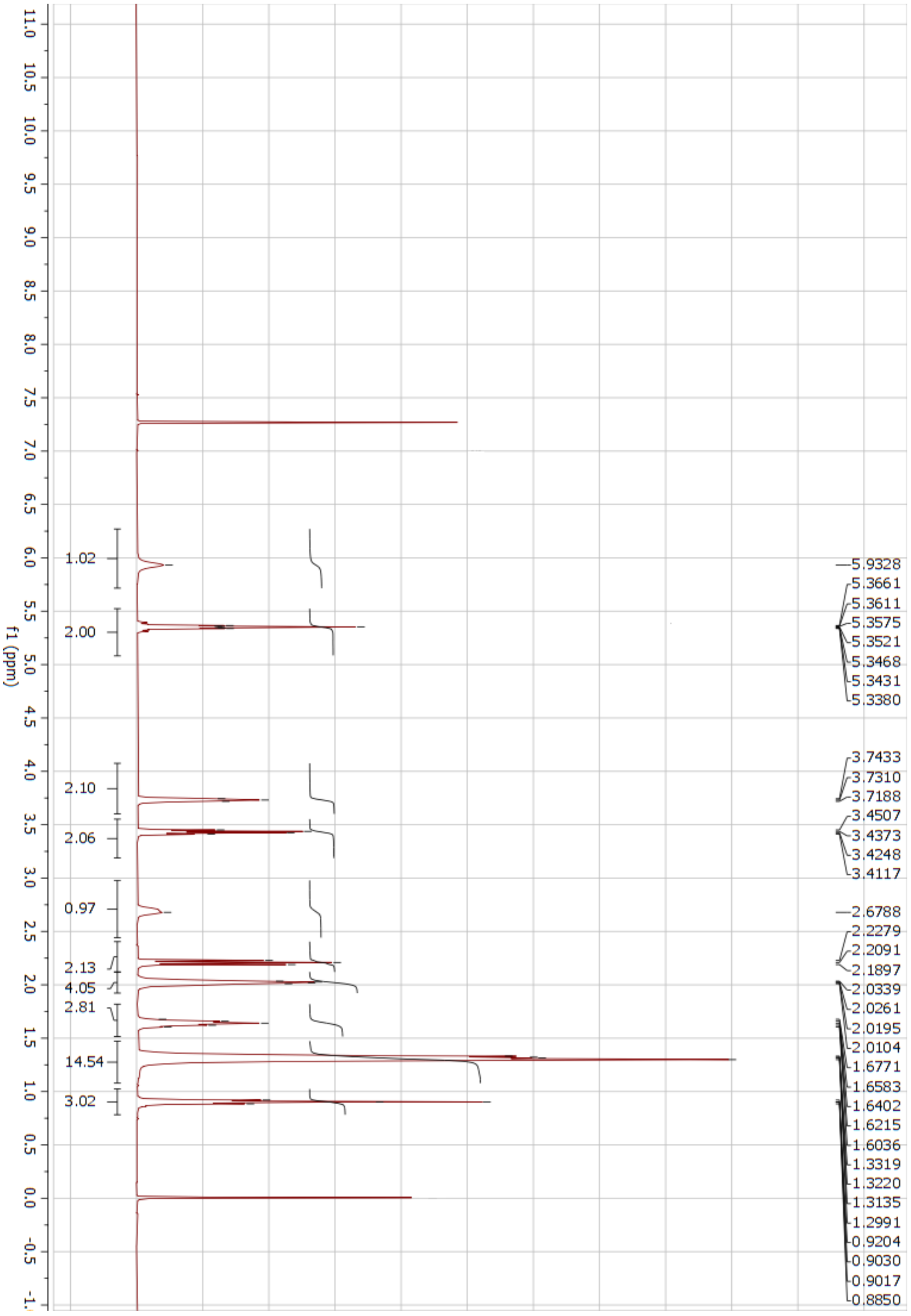

(Z)-*N*-(2-hydroxyethyl)hexadec-9-enamide (8)

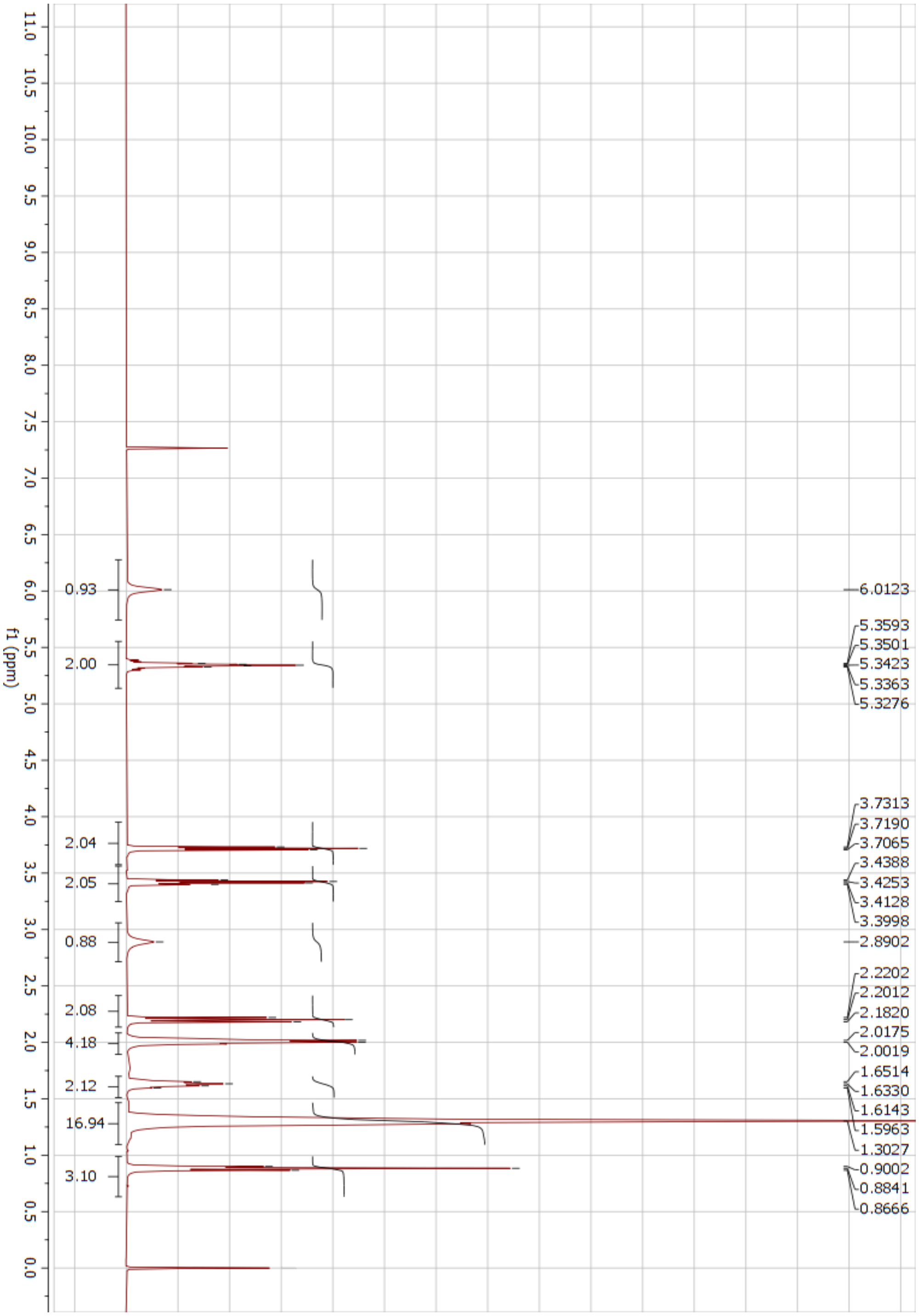

(Z)-*N*-(2-hydroxyethyl)heptadec-10-enamide (9)

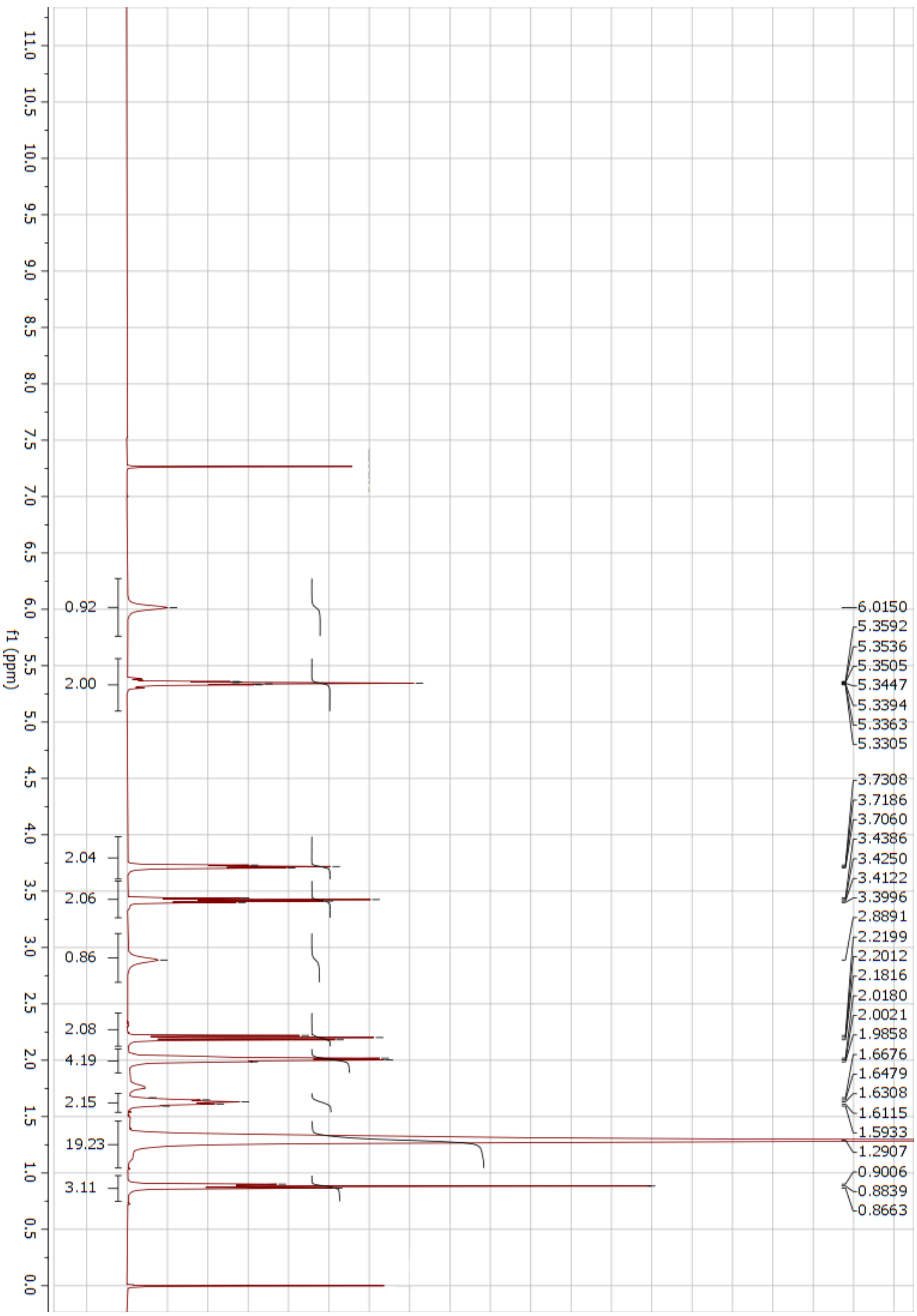

(Z)-*N*-(2-hydroxyethyl)octadec-9-enamide (OEA - 10)

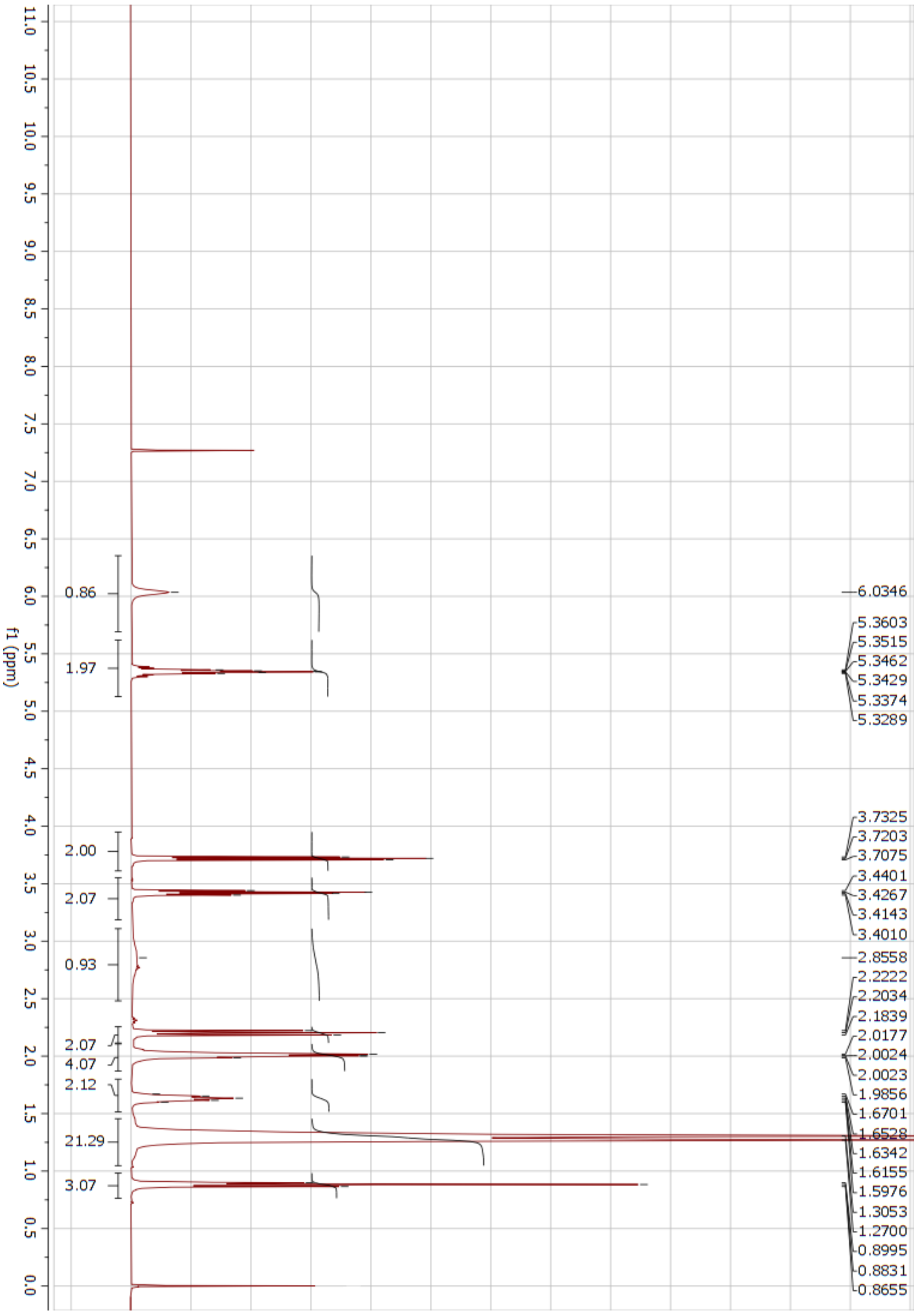

(Z)-*N*-(2-hydroxyethyl)nonadec-10-enamide (11)

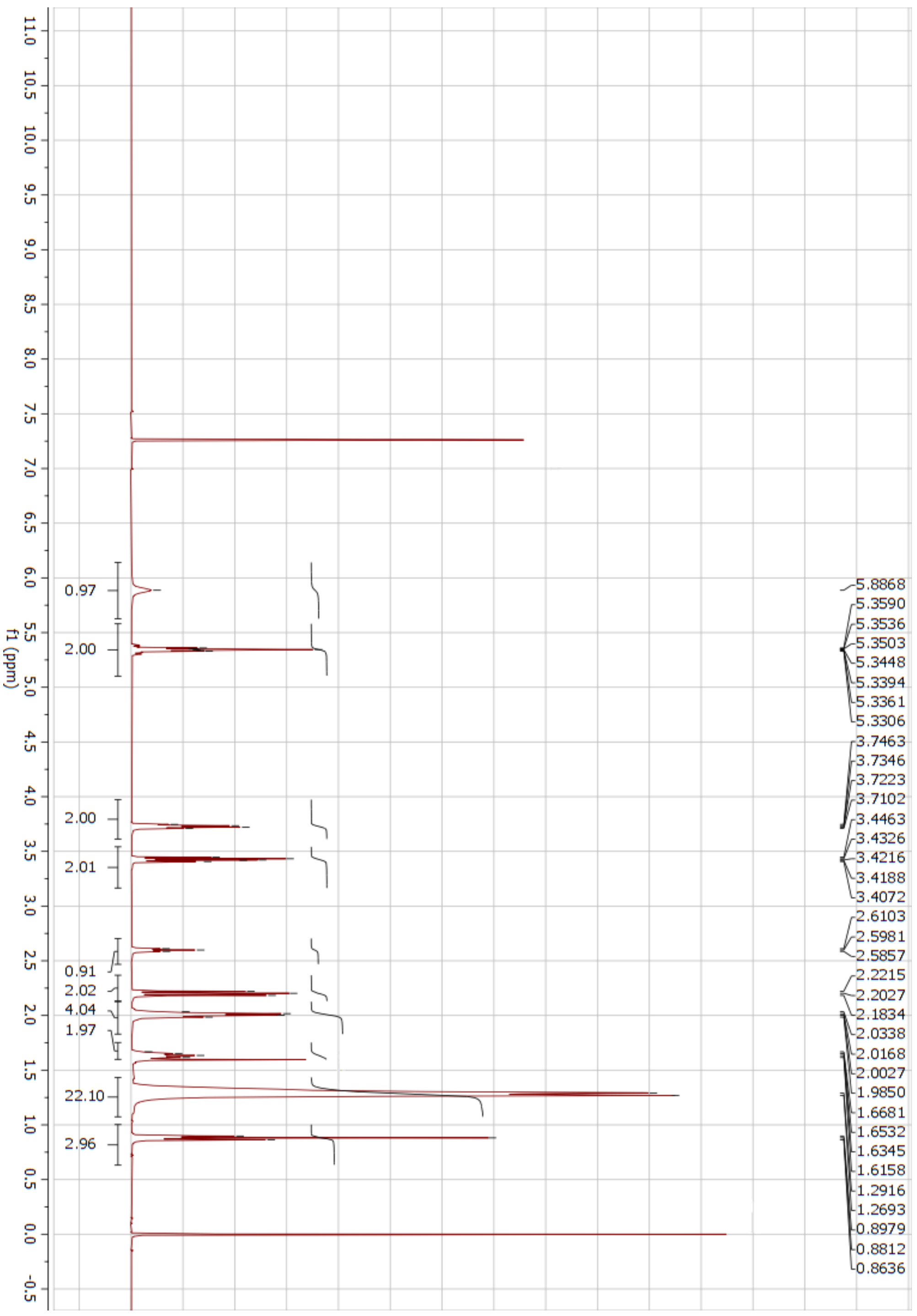

(Z)-*N*-(2-hydroxyethyl)eicos-9-enamide (12)

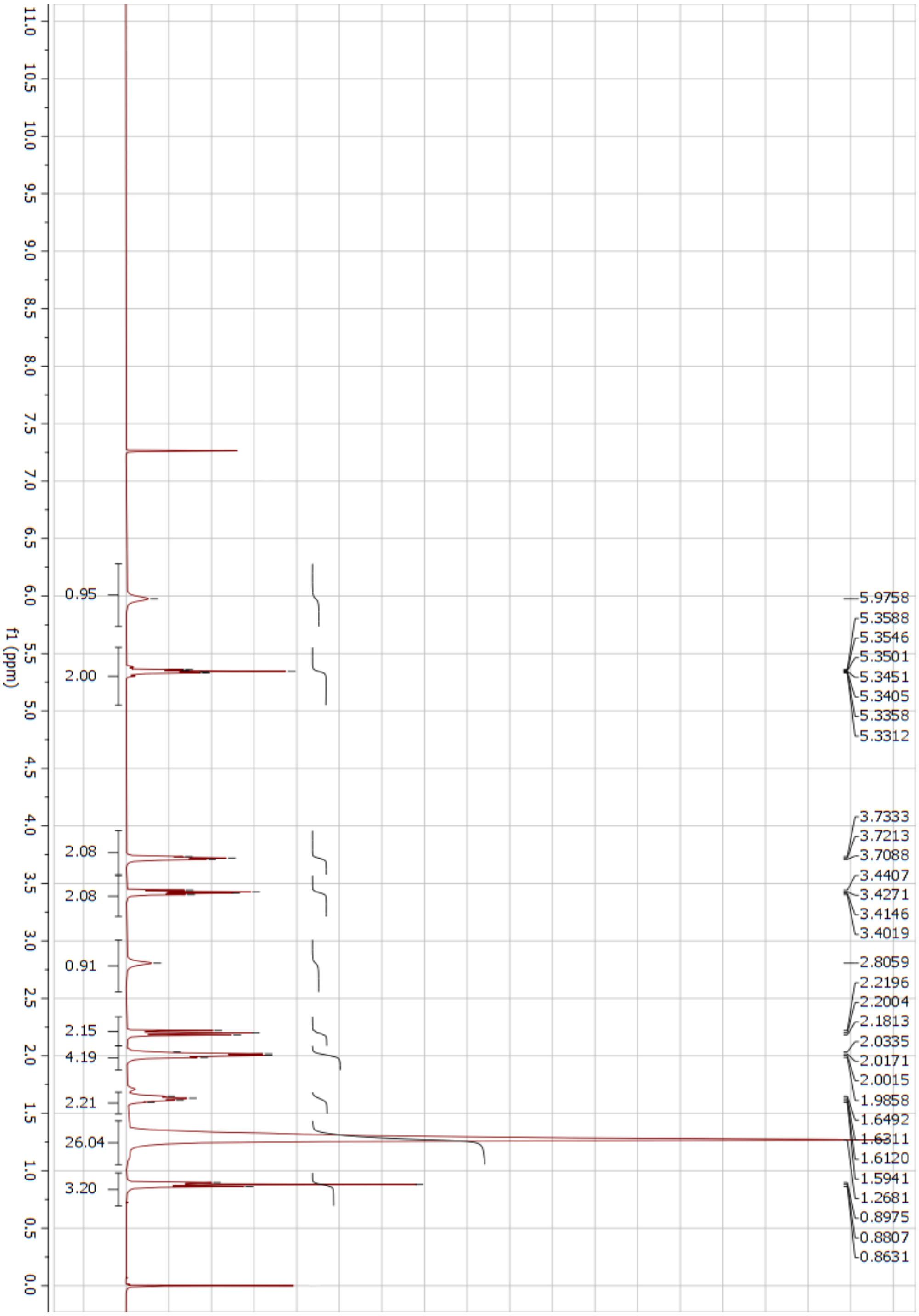

Palmitaminde (13)

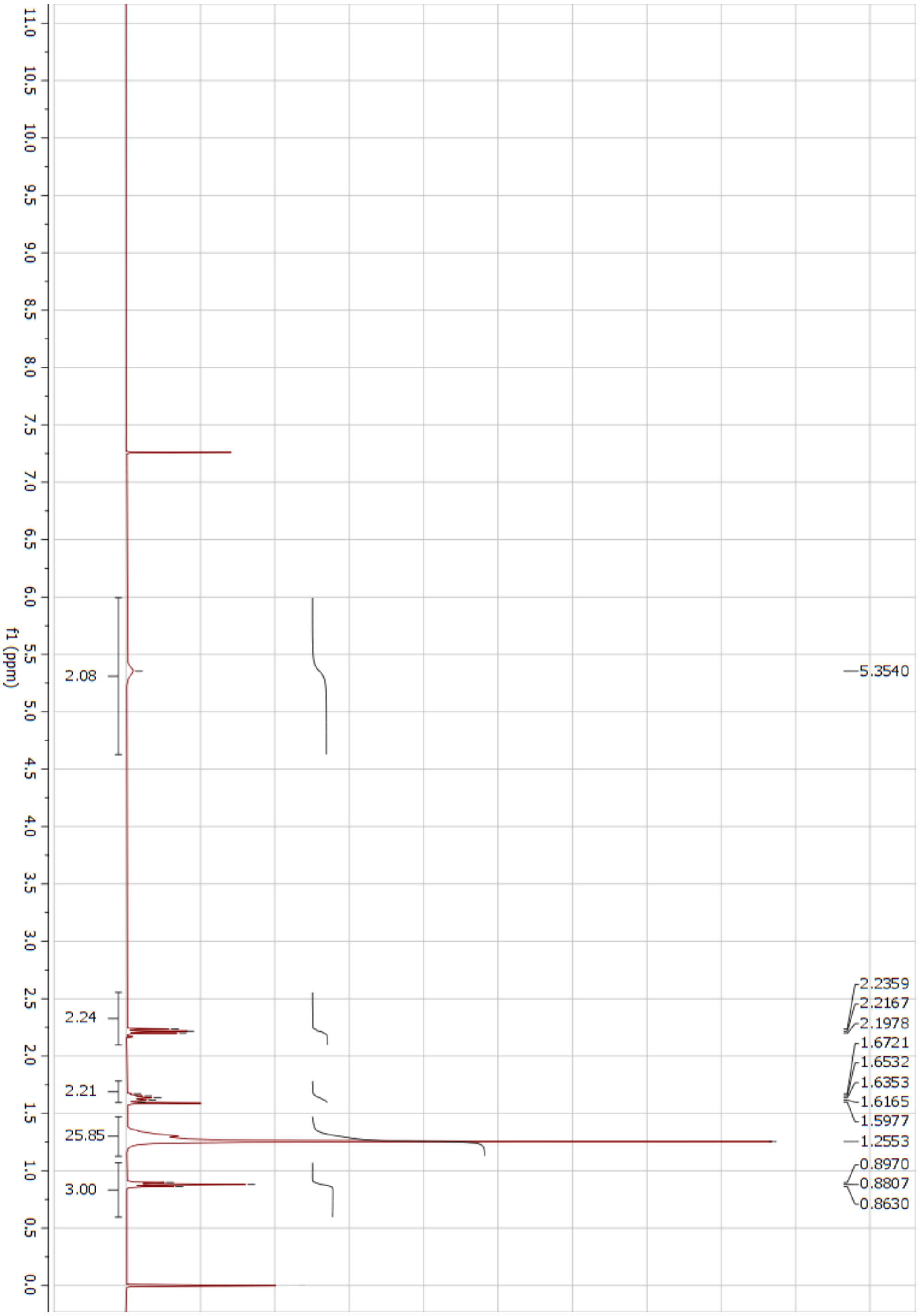

***N*-Methylpalmitamide (14)**

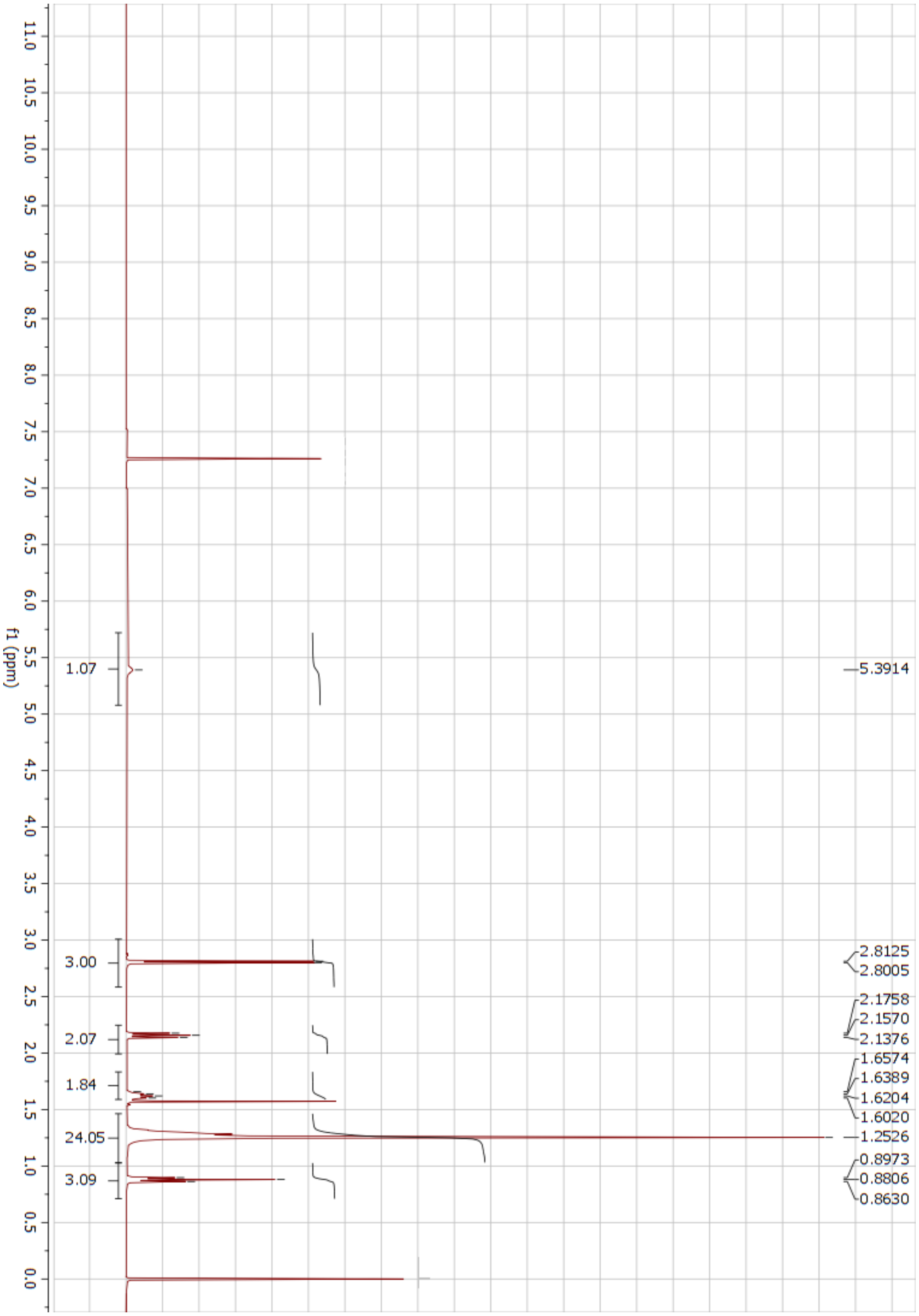

(S)-*N*-(1-hydroxypropan-2-yl)palmitamide (15)

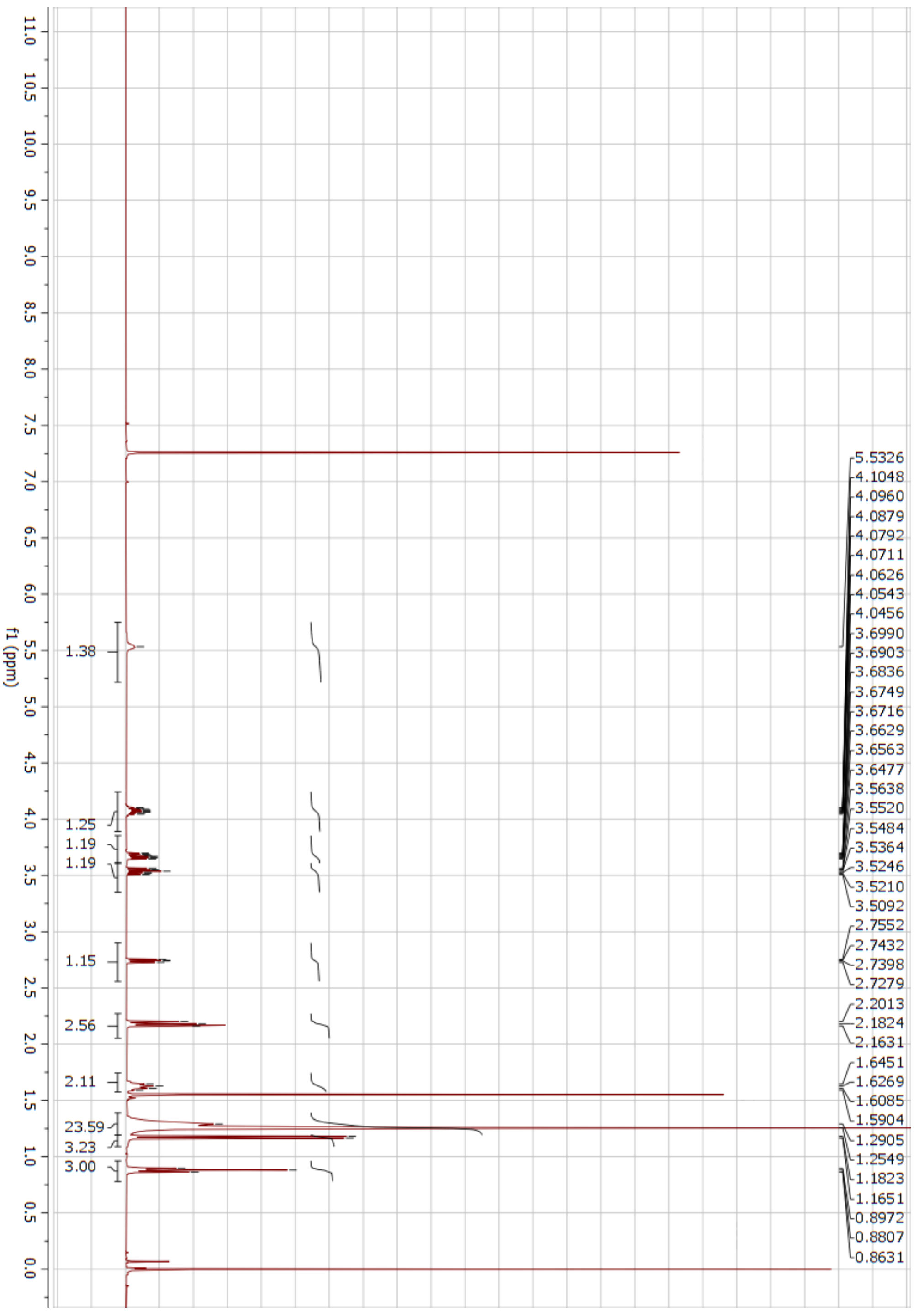

(R)-*N*-(1-hydroxypropan-2-yl)palmitamide (16)

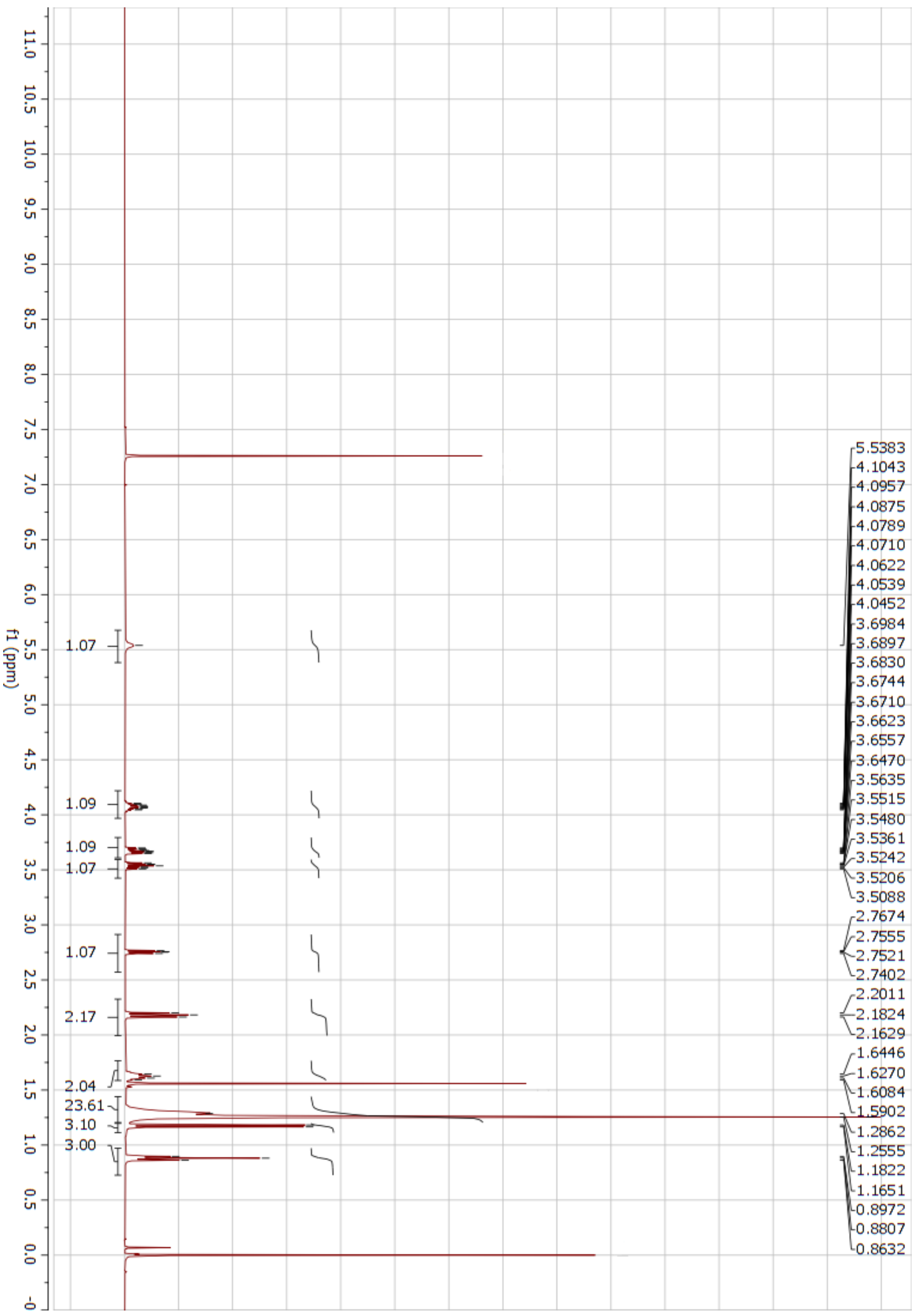

Supplement: Supplemental Material [file IENZ_A_1912035_SM5086.pdf]
